# Cycloheptatrienyl trianion: an elusive bridge in the search of exchange coupled dinuclear organolanthanide single-molecule magnets†
†Electronic supplementary information (ESI) available: Full synthetic details, crystallography, magnetic properties measurements, and *ab initio* details. CCDC 1454168–1454171. For ESI and crystallographic data in CIF or other electronic format see DOI: 10.1039/c6sc01224h



**DOI:** 10.1039/c6sc01224h

**Published:** 2016-08-08

**Authors:** Katie L. M. Harriman, Jennifer J. Le Roy, Liviu Ungur, Rebecca J. Holmberg, Ilia Korobkov, Muralee Murugesu

**Affiliations:** a Department of Chemistry and Biomolecular Sciences , University of Ottawa , Ontario , Canada K1N 6N5 . Email: m.murugesu@uottawa.ca ; Fax: +1-613-562-5170 ; Tel: +1-613-562-5800 ext. 2733; b Theory of Nanomaterials Group , INPAC – Institute of Nanoscale Physics and Chemistry , Katholieke Universiteit Leuven , Celestijnenlaan 200F , 3001 Leuven , Belgium; c Division of Theoretical Chemistry , Lund University , Getingevagen 60, P. O. Box 124 , 22100 , Lund , Sweden

## Abstract

The preparation of η-cyclopentadienyl (η^5^-C_5_R_5_), η-arene (η^6^-C_6_R_6_), and η-cyclooctatetraenyl (η^8^-C_8_R_8_) bridging motifs are common in organometallic chemistry; however, the synthetic preparation of η-cycloheptatrienyl (η^7^-C_7_R_7_) bridging motifs has remained a synthetic challenge in 4f chemistry. To this end, we have developed a synthetic route towards a series of rare dinuclear organolanthanide inverse sandwich complexes containing the elusive η^7^-C_7_H_7_ bridge. Herein, we present the structures and magnetic properties of the lanthanide inverse sandwich complexes [KLn_2_(C_7_H_7_)(N(SiMe_3_)_2_)_4_] (Ln = Gd^III^ (**1**), Dy^III^ (**2**), Er^III^ (**3**)) and [K(THF)_2_Er_2_(C_7_H_7_)(N(SiMe_3_)_2_)_4_] (**4**). These compounds are the first single-molecule magnets (SMMs) to feature this type of bridging motif. Furthermore, η^7^-C_7_H_7_ was found to efficiently promote ferromagnetic exchange interactions between metal ions. Variable temperature dc magnetic susceptibility measurements and subsequent simulations give significant exchange constants of *J* = +1.384, +1.798, and +3.149 cm^–1^ and dipolar constants of *J* = –0.603, –0.601, and –0.475 cm^–1^ for compounds **2–4**, respectively. Frequency dependent ac susceptibility measurements under an applied static field resulted in the observation of dual relaxation processes, and brought forth a greater understanding of the intermolecularly driven process at high frequency. In particular, this type of analysis of compound **3** under 800 Oe elicited an energy barrier of *U*_eff_ = 58 K. *Ab initio* calculations were performed in order to understand the nature of magnetic coupling and the origin of slow relaxation of magnetisation. Through these studies, the effect of the amido ancillary ligands on the magnetic axiality of the lanthanide ions was found to be competitive with the crystal field of the η^7^-C_7_H_7_ π-electron cloud. Our findings suggest that the tunability of the dipolar and exchange components of the magnetic interactions lie within the dihedral angle imposed by the amido ligands, thus offering potential for the development of new exchange coupled lanthanide systems.

## Abstract 2

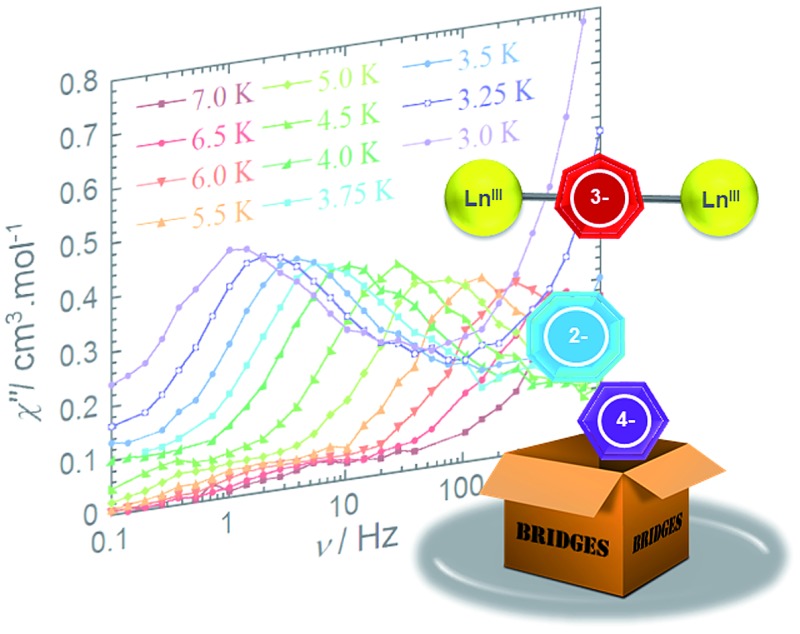
Lanthanide inverse sandwich compounds of the cycloheptatrienyl trianion give rise to ferromagnetic exchange and slow relaxation of the magnetisation.

## Introduction

**Chart 1:**
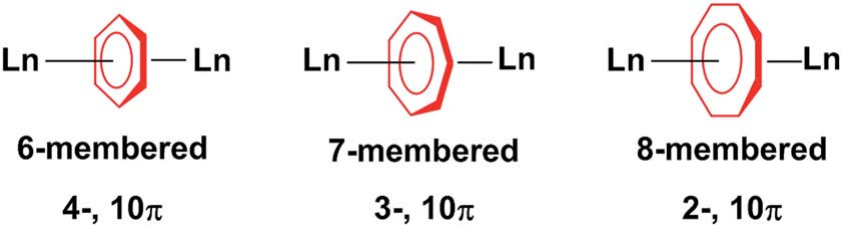
Ln^III^ ions bridged by 6-, 7- and 8-membered rings.

The use of η-cyclopentadienyl (η^5^-C_5_R_5_), η-arene (η^6^-C_6_R_6_) and η-cyclooctatetraenyl (η^8^-C_8_R_8_) ligands in the synthesis of organolanthanide complexes is widespread. These complexes have been extensively studied for their unique physical properties arising from their core 4f orbitals. While the cycloheptatrienyl trianion was first spectroscopically characterised by Bates *et al.* in 1977;^1^ only a single example of a η^7^-C_7_R_7_ lanthanide complex is known.^2^ There are only four reported examples of the isolation of f-element compounds with η-cycloheptatrienyl,^2–4^ and only two of those reports describe their use in dinuclear systems.^2,4^ The limited exploration of such species resides in the difficulty of the synthetic preparation and isolation of the elusive 10π-electron 7-membered ring with f-elements (Chart 1).

The amplified interest in the isolation of dinuclear lanthanide complexes with different bridging motifs arises from the ability to probe magnetic communication between metal ions, as they represent the most fundamental unit with which to study magnetic exchange interactions. Some of these reported molecules exhibit slow relaxation of magnetisation below their blocking temperature; these molecules are termed Single-Molecule Magnets (SMMs).^5–11^ Since the discovery of the first organometallic SMMs in 2010,^12^ several complexes have been reported to exhibit this nanomagnetic behavior.^13–17^ Most of the examples described are mononuclear compounds, however, the study of dinuclear systems is of the utmost importance when we consider the technological requirements of the future. In regard to SMMs, these include increasing the total spin of molecular magnets through expanding the number of paramagnetic centres. While many different bridging systems exist,^14,18,19^ few examples have demonstrated the importance of planar aromatic organometallic ligands towards garnering favourable magnetic interactions.^20–22^ These systems are an appealing design strategy as they may be employed as building blocks to generate higher nuclearity compounds, while they are more notably effective in harnessing the inherent magnetic anisotropy of 4f and 5f ions.^13,15–17,23,24^ Our recent reports with cyclooctatetraenyl^20,21^ and arene-bridged^22^ systems shows that a weak, yet non-negligible, interaction can be observed with coupling constants between Gd^III^ ions of –0.644 cm^–1^ and –0.488 cm^–1^ respectively, utilizing the isotropic spin Hamiltonian (*H* = –2*JS*_a_ *S*_b_, *S*_a_ = *S*_b_ = 7/2) for each system respectively. Herein, the role of the 7-membered cycloheptatrienyl ring in the magnetic exchange between lanthanide ions will be examined and compared with its counterparts, the 6- and 8-membered rings (Chart 1). We investigate how subtle structural differences in a family of rare inverse sandwich compounds influence the overall magnetic properties and clearly demonstrate the significance of the 7-membered ring on the bridging interactions and magnetic axiality. We report for the first time Gd^III^, Dy^III^, and Er^III^ compounds with the cycloheptatrienyl bridge. The synthesis, structure, and magnetic characterization of three isostructural dinuclear complexes, [KLn_2_(C_7_H_7_)(N(SiMe_3_)_2_)_4_] (Ln = Gd^III^ (**1**), Dy^III^ (**2**), Er^III^ (**3**)) and one structurally analogous complex, [K(THF)_2_Er_2_(C_7_H_7_)(N(SiMe_3_)_2_)_4_] (**4**) is presented.

## Results and discussion

### Syntheses and structures

Since the first report of the synthesis of a uranium cycloheptatrienyl sandwich complex in 1995,^3^ there has been limited exploration into the isolation of other f-element complexes containing cycloheptatrienyl. However, other areas of chemistry, such as organic chemistry, have made use of the 6π-electron cycloheptatrienyl cation (the tropylium ion),^25^ and there have been reports of the 10π-electron derivative in transition metal chemistry.^26^ Thus, the preparation of the above mentioned complexes, **1–4**, was carefully designed to result in the facile formation of the trianion through employing chemistry that is previously known for lanthanide ions. In particular, this chemistry involves the polarisation of C–H bonds,^27^ and is further complemented by highly basic and sterically demanding ancillary ligands.

**Scheme 1:**
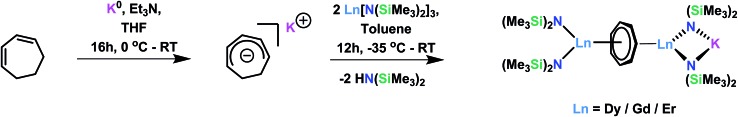
Synthesis of [KLn_2_(C_7_H_7_)(N(SiMe_3_)_2_)_4_]” (Ln = Dy^III^, Gd^III^, Er^III^).

Inspired by the work of Arliguie *et al.*,^4^ who had utilized borohydride chemistry towards the isolation of an f-element η^7^-C_7_H_7_ complex, we attempted to utilize lanthanide borohydrides to support the inverse sandwich architecture. However, due to the highly reactive/reducing nature of the borohydrides and the non-innocent character of the cycloheptadienide ligand, the isolation of such systems proved to be difficult. In order to combat the aforementioned issue, we employed bis(trimethylsilyl) amido ancillary ligands and have since prepared a series of dinuclear complexes of Ln = Dy^III^, Gd^III^, Er^III^ (Scheme 1). The synthesis of Ln^III^[N(SiMe_3_)_2_]_3_ was first reported by Bradley *et al.*,^28,29^ and has since been revisited in order to investigate the SMM properties of the complexes, which arise from their distinctive crystal field.^30^ Conversely, the seven-membered bridging motif may be prepared from the commercially available 1,4-cycloheptadiene, where upon a one-electron reduction with potassium metal in the presence of Et_3_N, cycloheptadienide (C_7_H_9_^–^) (Scheme 1) is afforded. The salt, KC_7_H_9_, remains stable for several days under inert conditions at –35 °C.

Solutions of lanthanide tris(bis(trimethylsilyl) amido) and potassium cycloheptadienide are combined at –35 °C in toluene and warmed to room temperature gradually. Further reduction of the cycloheptadienide to the aromatic trianion, cycloheptatrienyl, is supported by a mechanism previously reported by Miller and Dekock.^27^ Initial coordination of the Ln^III^ ion results in polarisation of the methylene C–H bond and subsequent proton abstraction by a strong base. Interestingly, it was first postulated that the highly basic nature of the C_7_H_9_^–^ may be responsible for this abstraction, resulting in the formation of 1,4- and 1,3-isomers of cycloheptadiene. However, in this case, the loss of an amido ligand from each of the bridging Ln^III^ ions may suggest that abstraction occurs *via* the amido, thus inducing the generation of soluble HN(SiMe_3_)_2_ species. The presence of such species was observed in the crude ^1^H NMR of compound **3** as a singlet at 0.1 ppm in toluene-d_8_ at 298 K, further supporting this hypothesis.

**Fig. 1:**
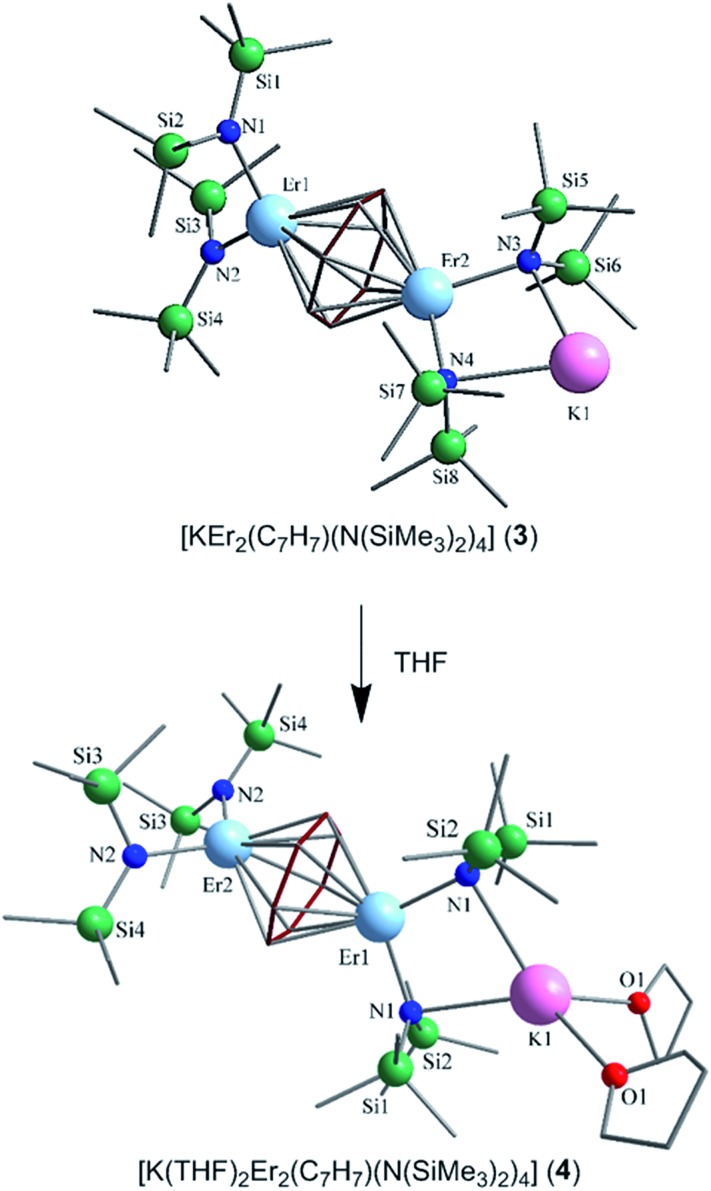
SCXRD structures for compounds **3** (top) and **4** (bottom). The cycloheptatrienyl ligand is displayed in dark red. Colour code: light blue (Er^III^), pink (K), green (Si), blue (N), red (O), grey (C). H atoms have been omitted for clarity.

Nevertheless, collection of the filtrate followed by treatment with toluene and hexanes yields compounds **1–3**. Conversely, the solvated derivative, compound **4**, can be obtained from **3** *via* extraction into THF (Fig. 1), resulting in the coordination of two molecules of THF to the bound potassium ion, and thereby limiting intermolecular interactions. X-ray quality crystals of **4** were isolated from the subsequent treatment with a toluene/hexanes mixture, confirming the nature of the solvated species.

Single-crystal X-ray diffraction (SCXRD) studies reveal that compounds **1–3** are isostructural and crystallize in the monoclinic space group *P*2_1_/*n*. On the other hand, the analogous compound **4** crystallizes in the monoclinic space group *C*2/*c*. The structure of the Er^III^ analogue, **3**, will be the representative structure described herein (Fig. 1, top). The molecular structure of **3** reveals an inverse cycloheptatrienyl sandwich complex. The dinuclear compound is composed of two Er^III^ ions bridged by the 10π-electron cycloheptatrienyl C_7_H_7_^3–^ trianion in a η^7^-bound fashion, with an Er–C bond distance range of 2.484(8)–2.629(9) Å. The remaining coordination environment is occupied by two [N(SiMe_3_)_2_]^–^ ligands. Interestingly, one K ion is bound to one side of the molecule *via* N atoms (N3, N4) from the [N(SiMe_3_)_2_]^–^ ligands, thus making this dinuclear unit unsymmetrical. Due to this binding configuration, the N3···Er2···N4 angle of 98.6(2)° is much smaller than the N1···Er1···N2 angle of 105.7(2)°. It is noteworthy that in the case of **4**, due to crystal packing effects the symmetry of the molecule is slightly higher than in **3**.

Close inspection of the packing arrangement of **3** reveals a close contact between the K ion and a carbon atom (C14) from the [N(SiMe_3_)_2_]^–^, which subsequently promotes a linear chain-like arrangement of the molecules (Fig. S4†). Interestingly, in the case of compound **4** we still observe a head-to-tail packing arrangement generating a chain-like array, however, there are no close contacts that exist beyond H–H interactions (Fig. S5†).^31^ In regard to compound **3**, the intramolecular Er–Er distance of 3.9580(7) Å is slightly shorter than the distance observed in a COT″ (1,4-bis(trimethylsilyl)cyclooctatetraenyl dianion) bridged Er_2_ dimer (4.1109(5) Å) or an arene bridged Er_2_ compound (4.067(1) Å).^21,22^ A similar Sm_2_ inverse sandwich analogue was reported with a bridging COT and terminal [N(SiMe_3_)_2_]^–^ ligands with a Sm–Sm distance of 4.308(1) Å.^32^ However, the larger distance in the case of the Sm example is primarily due to the larger ionic radii of the Sm^III^ ion. Finally, it is noteworthy that the Nd^III^ analogue of the reported example exhibits a Nd–Nd distance of 4.213(3) Å, this is presumably a result of the electron rich borohydride ancillary ligands, which allow for increased electron donation to the electropositive Nd^III^ ions.^2^

The central cycloheptatrienyl ligand adopts a planar geometry, owing to its 10π-electron aromatic configuration, with the largest atom deviation being 0.06 Å out of the plane formed by the seven C atoms. The high charge (–3) and planarity of the bridging ligand, along with the close proximity of Er^III^ ions, is expected to lead to non-negligible magnetic interactions *via* the delocalised π-orbitals of the cycloheptatrienyl ligand. Therefore, this molecule represents an ideal candidate to probe the exchange interactions between metal ions, while also studying the ligand field effects of the bridging unit in comparison with its COT and arene counterparts.

### Static magnetic properties

**Fig. 2:**
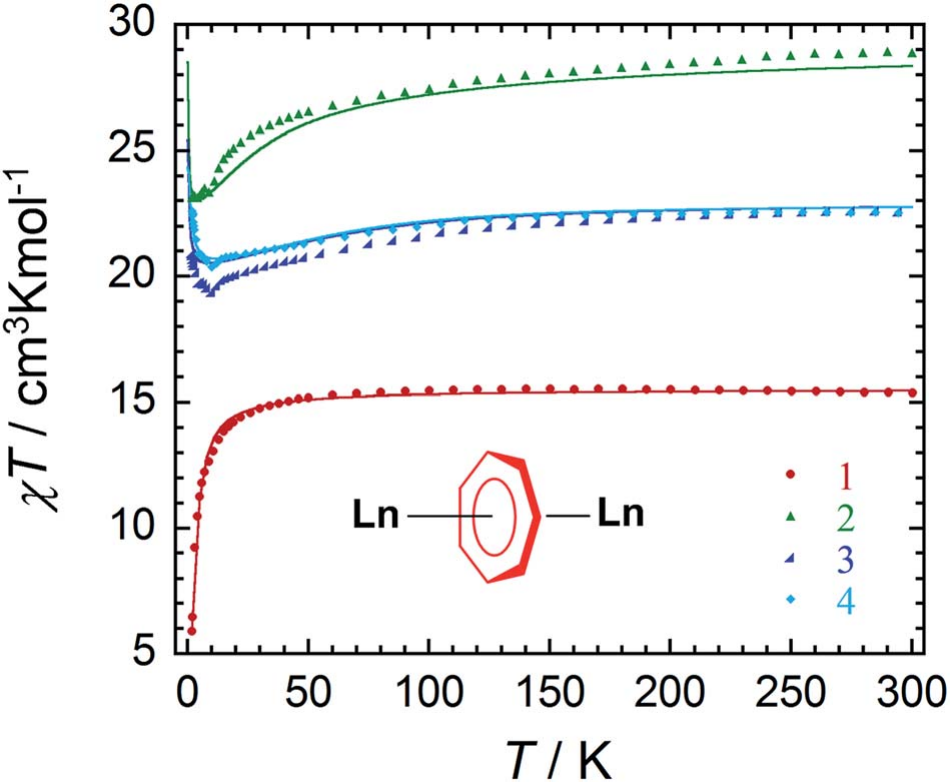
Temperature dependence of the *χT* product at 0.1 T for compound **1** (), **2** (▲), **3** (), and **4** (♦), with *χ* being the molar magnetic susceptibility per molecule defined as *M*/*H*. The solid line for **1** represents the fit as determined from the application of the 2*J* formalism. The solid lines for **2–4** correspond to *ab initio* calculated magnetic susceptibilities, using the method described in the text. The calculated susceptibility for **2** has been scaled by +2.5%.

Direct current (dc) and alternating current (ac) magnetic susceptibility measurements were performed using a SQUID magnetometer on crushed crystalline samples of complexes **1–4**, prepared under an inert atmosphere. Variable temperature magnetic susceptibility measurements under a 0.1 T applied field in the temperature range of 1.9–300 K are shown in Fig. 2. At room temperature, the *χT* values of complexes **1–4** are 15.37, 28.34, 22.54 and 22.49 cm^3^ K mol^–1^, respectively. These values are in good agreement with the expected theoretical values of 15.76 (Gd^III^: ^8^S_7/2_, *S* = 7/2, *L* = 0, *g*_*J*_ = 2), 28.34 (Dy^III^: ^6^H_15/2_, *S* = 5/2, *L* = 5, *g*_*J*_ = 4/3) and 22.49 cm^3^ K mol^–1^ (Er^III^: ^4^I_15/2_, *S* = 3/2, *L* = 6, *g*_*J*_ = 6/5) for two non-interacting lanthanide ions. For **1**, the *χT* product remains constant down to 50 K, followed by a gradual decrease with temperature to reach a minimum value of 5.84 cm^3^ K mol^–1^ at 1.9 K. This downturn of the *χT* product can be attributed to the intramolecular antiferromagnetic interactions between the spin carriers (4.0869(7) Å). Owing to the isotropic nature of Gd^III^ ions, the strength of interactions between the two lanthanide ions can be quantified. Application of the Van Vleck equation to the Kambe's vector coupling method was completed by using the isotropic spin Hamiltonian *H* = –2*JS*_a_ *S*_b_, with *S*_a_ = *S*_b_ = 7/2, which was used to fit the variation of *χT vs. T*. The best-fit yielded a *J* value of –0.134 cm^–1^ for compound **1**. The obtained *J* value is rather weak as a consequence of the shielded f-orbitals of Gd^III^ having minimal orbital overlap with the bridging ligand.

In comparison with the 6- and 8-membered rings, the obtained coupling constant for Gd^III^ is slightly smaller, and unfortunately did not lead to a direct trend related to ring size and charge density. When considering both the dipolar and exchange contributions to the coupling, as determined by *ab initio* methods (*vide infra*), both components for the 7-membered ring remain the smallest of any of the computed parameters. Perhaps an explanation for this lies within the ligand field contributions from the ancillary ligands. This may be considered from a formal charge perspective, such that the cycloheptatrienyl bridge adopts a formal charge of –3, which, when distributed over seven atoms, is diluted to approximately –0.43 per C atom. Conversely, the charge distribution over the amido N atom remains highly concentrated. Thus the interaction with the amido ligands remains dominant (*vide infra*) compared to the donating ability of the bridging C_7_-moiety. This was further proven through our computational studies of the main magnetic axis and LoProp charges (*vide infra*). Lastly, the presence of the potassium ion prevents the Ln^III^ ions from receiving equal electronic donation from the amido ancillary ligands, where the electron density of N3 and N4 would be split between Er2 and K1, thereby making Er2 less electron rich in comparison to Er1.

In the case of the anisotropic compounds **2–4**, the *χT* profile differs significantly from the Gd^III^ analogue. For example, the *χT* product of compound **2** decreases very slowly from 300 K with temperature, followed by a more rapid decrease below 20 K to reach a minimum value of 23.15 cm^3^ K mol^–1^ at 1.9 K. On the other hand, the *χT* product of compounds **3** and **4** exhibit a slightly different trend upon decreasing temperature. The *χT* products for compounds **3** and **4** decrease gradually from 300 K to minimum values below 15 K of 19.64 cm^3^ K mol^–1^ and 20.53 cm^3^ K mol^–1^, respectively. This decrease in the *χT* product is followed by a rapid increase below 10 K to reach maximum values of 20.92 cm^3^ K mol^–1^ and 22.58 cm^3^ K mol^–1^, respectively. The final increase in the value of the *χT* product is attributed to intramolecular ferromagnetic interactions between the Er^III^ ions. This will be further confirmed through *ab initio* calculations (*vide infra*).

As seen in Fig. S6–S9,† the field dependence of the magnetisation measurements performed at low temperatures exhibit non-saturation, even at 7 T and 1.8 K, for all compounds. This can be attributed to weak intramolecular antiferromagnetic interactions between the Ln^III^ ions, thereby making the low lying excited states accessible by applying a magnetic field, even at the lowest measurable temperature of 1.8 K. This finding is further exemplified through our computational study, where the energies of the first and second excited states are minimally separated from the ground state (*vide infra*). In the case of compounds **2–4** the presence of magnetic anisotropy is also likely to contribute to this lack of saturation in the magnetisation. Contrary to the COT″ bridged counterparts, no hysteretic behaviour was observed down to 1.8 K and therefore alternating current (ac) magnetic susceptibility measurements were performed to investigate the potential SMM behaviour of the anisotropic compounds **2–4**.

### Dynamic magnetic properties

**Fig. 3:**
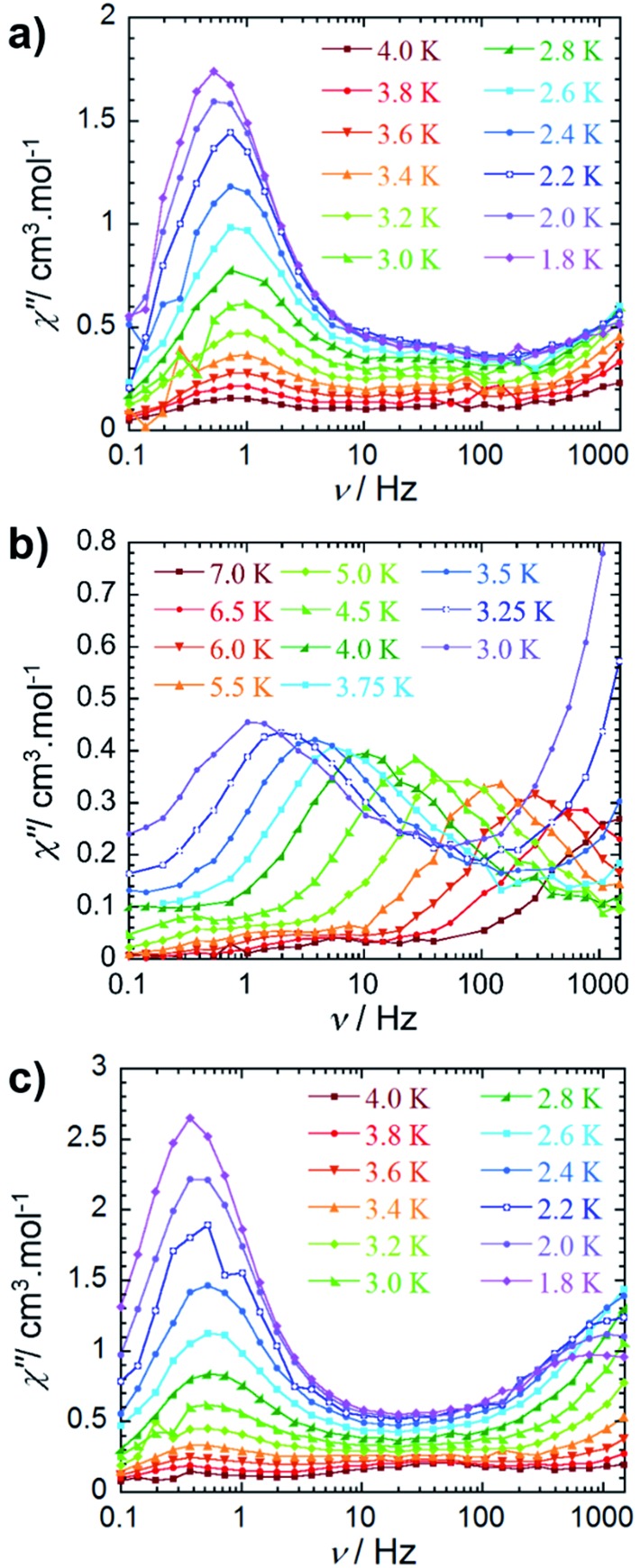
Frequency dependence of the out-of-phase (*χ*″) ac susceptibility (a) for **2** under *H*_dc_ = 2000 Oe, (b) for **3** under *H*_dc_ = 800 Oe and (c) for **4** under *H*_dc_ = 2000 Oe between 0.1 and 1000 Hz, at indicated temperatures. See the ESI† for data collected under *H*_dc_ = 1000 Oe for compound **4**.

An ac field of 3.78 Oe was utilized to probe the slow relaxation dynamics of compounds **2–4**, however, no ac signal was observed at zero applied dc field for all compounds. This is common for lanthanide systems with significant quantum tunnelling of magnetisation (QTM). However this QTM can be minimised upon application of a static dc field. As such, a frequency dependent signal was observed for all three compounds (Fig. 3 and S10–S12†) with the application of an optimised dc field. With respect to compound **2**, the application of an optimal dc field of 2000 Oe allowed for the observation of a low frequency process below 4 K. The out-of-phase susceptibility of this processes exhibited minimal shifting in peak maxima with regards to frequency upon decreasing temperature. This type of behaviour may be indicative of a dominant QTM regime. This is not surprising due to the potential for low lying exchange coupled states, thereby enabling a shortcut in the energy barrier such that the first excited exchange state lies only minimally above the ground state with a calculated energy of 1.9 × 10^–5^ cm^–1^ (*vide infra* Table 2). However, we cannot rule out the possibility of intermolecular interactions, as application of large static fields has been shown to propagate spin–spin interactions.^33,34^ These types of interactions may lead to the formation of magnetic domains, consequently precluding the analysis from a molecular perspective. Due to these phenomena, an effective energy barrier for this process could not be extracted from this data set. Alternatively, a frequency-dependent *χ*″ signal was observed under a static dc field for **3** (Fig. 3b). The lack of overlapping peak maxima at low temperatures suggests that QTM is minimized with the application of an optimal static field of 800 Oe.

**Table 1:** Electronic and magnetic properties of the individual metal sites in compounds **2–4**, obtained from *ab initio* calculations (in cm^–1^)

**2**		**3**		**4**	
Dy1	Dy2	Er1	Er2	Er1	Er2
0	0	0	0	0	0
116	69	122	74	68	88
328	270	148	138	136	149
530	441	211	188	182	199
720	614	262	231	228	249
851	754	334	285	289	332
1010	854	525	476	476	523
1214	1021	573	501	508	589

**Main values of the *g* tensor in the ground Kramers doublet**					
0.008	0.027	0.016	0.013	0.007	0.019
0.020	0.089	0.039	0.057	0.049	0.064
18.825	18.370	17.763	17.978	17.883	17.782

**Angle with the N–Ln–N plane (degrees)**					
1.3	1.3	87.0	88.6	89.3	90.0

**Table 2:** Exchange coupled states and their magnetic anisotropy in compounds **2–4**, employing the reported coupling parameters. Exchange and dipolar coupling parameters are given with respect to eqn (1)

**2**	**3**	**4**
*J*_dip_ = –0.603	*J*_dip_ = –0.601	*J*_dip_ = –0.475
*J*_exch_ = +1.384	*J*_exch_ = +1.798	*J*_exch_ = +3.149

**Low-lying exchange coupled states (cm** ^**–1**^ **)**		
0.000000	0.000000	0.000000
0.000019	0.000032	0.000077
0.391064	0.598359	1.337034
0.391092	0.598405	1.337149
69.220130	74.152765	68.455939
69.220149	74.152981	68.461815
69.503274	74.260012	68.544184
69.503276	74.260236	68.550104

***g***_***Z***_ **values** ^*a*^ **in the ground and first excited exchange doublet states**		
29.8	28.5	27.9
22.3	21.6	22.4

^*a*^ *g*_*X*_ = *g*_*Y*_ = 0 for Ising doublets, according to the Griffith's theorem;^62^ (*i.e.* for systems with even number of electrons).

Observation of the shifting of peak maxima to lower frequencies below 7 K demonstrates the presence of slow relaxation of magnetisation in **3**, indicating field-induced slow relaxation. From the *χ*″ data measured between 7 and 3 K, the Arrhenius law (*τ* = *τ*_0_ exp(*U*_eff_/*kT*)) was employed in order to extract an effective energy barrier of 58 K, and a pre-exponential factor of 2.9 × 10^–8^ s (Fig. S13†). More notably, the frequency dependent behaviour is mostly likely attributed to single-ion properties, as the observation of a second relaxation process at high frequencies becomes evident below 3.75 K. Full analysis of this process could not be completed due to the frequency limitations (0.1–1500 Hz) of the magnetometer. From a structural perspective, the observed single-ion behaviour of **3** is not surprising given the non-centrosymmetric nature of the molecule. Inequivalent metal ion sites have elicited dual relaxation processes at low temperatures in previous studies.^35–41^ However, with respect to compound **3**, this is easily visualized *via* the lack of an inversion centre within the molecule as a consequence of the coordinated potassium ion.

The observed magnetic behaviour of **3** greatly contrasts with the results obtained for the Dy^III^ analogue, **2**, suggesting that the cycloheptatrienyl trianion, along with the [N(SiMe_3_)_2_]^–^ ancillary ligands, provide a more suitable ligand field for Er^III^ ions. These findings strongly correlate with our previous studies on COT″ bridging ligands, where the zero field energy barrier was improved upon from 25 K for the Dy^III^ analogue to 306 K for Er^III^.^20,21^ Additionally, we further exemplified that the ligand field provided by the delocalised π-cloud promoted greater magnetic axiality in Er^III^ ions over Dy^III^ ions in single-ion sandwich complexes of COT.^42^ While this remains true of the delocalised π-cloud and Er^III^ ions in the present study, the effects of the amido ligands prove dominant over the cycloheptatrienyl, effectively generating greater magnetic axiality in **2** (*vide infra*). This is in accordance with previous studies, such that the axial orientation of highly charged negative donor atoms favour the oblate electron density of Dy^III^ ions.^43–48^

**Fig. 4:**
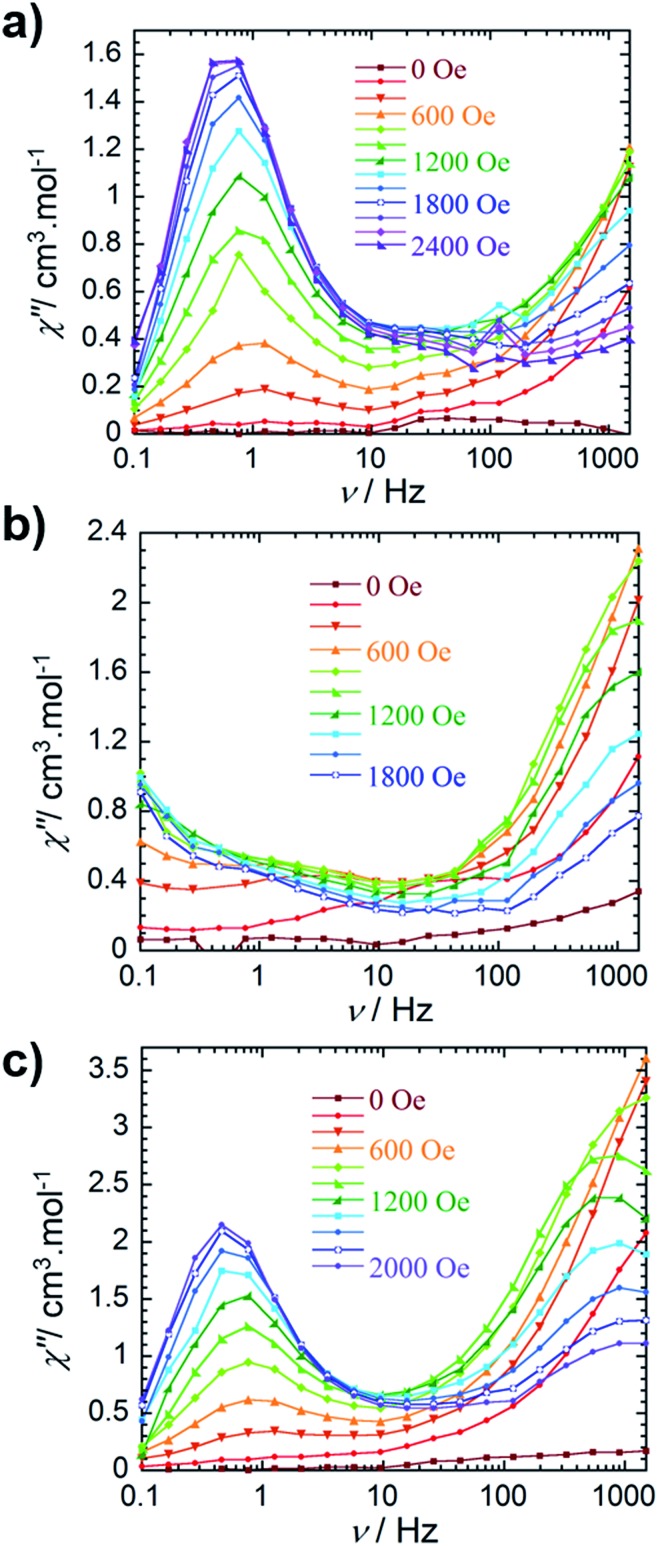
Frequency dependence of the out-of-phase (*χ*″) ac susceptibility collected at 2 K (a) for compound **2**, (b) for compound **3** and (c) for compound **4** at indicated dc fields.

The out-of-phase magnetic susceptibility of **4** reveals two independent relaxation processes below 4 K, similar to compound **3** (Fig. 3c and S11†). Once again this is not surprising given the unsymmetrical nature of the complex.^35–40^ In order to probe each of these processes, an optimal dc field of 1000 Oe was used to elucidate the nature of the high frequency process, whereas an optimal field of 2000 Oe was employed in the study of the low frequency process. Unfortunately, the nature of the collected data precluded the extraction of an energy barrier to spin reversal, however, it did prove fruitful in gaining a further understanding of the interactions occurring within this system. Interestingly, the low frequency processes exhibit similar characteristics to **2**, where upon decreasing temperature, the resulting out-of-phase signal increases in intensity, but demonstrates little-to-no frequency dependent behaviour. Again, this is most likely a result of the low-lying excited exchange states, which promote QTM. Our computational studies (*vide infra*) elucidated a first excited state energy of 7.7 × 10^–5^ cm^–1^ for compound **4** further supporting the nature of this process. Once more, it is worth noting that at large magnetic fields it becomes difficult to infer whether the observed properties are solely molecular in nature, due to the potential of induced spin–spin intermolecular interactions.^33,34^ Nonetheless, the presence of the secondary relaxation process at higher frequencies exhibits a shifting peak maxima towards lower frequency upon decreasing temperature (Fig. 3c). Interestingly, this plot is characterised by decreasing susceptibility intensity for an iso-temperature curve with decreasing temperature. This type of behaviour has been similarly noted in Single-Chain Magnets (SCMs), where inter-chain spin–spin interactions give rise to decreasing susceptibility values.^49,50^ Even under the optimal field of 1000 Oe, there is a decrease in intensity of the peaks for the out-of-phase component (Fig. S11†). While it is difficult to fully conclude the nature of the high frequency process, the preliminary data would suggest that the fundamental component relies on an intermolecularly driven process/relaxation. This finding may also explain the tails observed in the high frequency region of the out-of-phase susceptibility for compounds **2** and **3**. In fact, it is not uncommon in lanthanide-based systems to observe a secondary process as a result of intermolecular interactions.^34,51–53^ Further investigation into the frequency dependent ac susceptibility measurements as a function of dc field for **2–4** (Fig. 4), reveal an unusual field dependence in the second relaxation, such that the high frequency process appears to be augmented by weak static fields, this is likely a result of a direct relaxation process which is promoted by neighbouring spins^40,54^ thus supporting the proposed intermolecularly driven relaxation process.

### *Ab initio* studies

*Ab initio* calculations for **2–4** were performed in order to gain additional insight into the electronic and magnetic structures of these compounds. All calculations performed were CASSCF/RASSI/SINGE_ANISO,^55^ and employed SCXRD structural data. Electronic and magnetic properties of the individual Ln^III^ sites were obtained through fragment *ab initio* calculations. The calculated structures have identical structures to those obtained for complexes **2–4**, where the neighbouring lanthanide sites are computationally replaced by the diamagnetic Lu^III^. The CASSCF wavefunction includes all possible electron distributions within the 4f^9^ (for Dy^III^) and 4f^11^ (for Er^III^) shells only, while the remaining orbitals were kept doubly occupied. The orbitals and coefficients of the individual configurations were optimized self consistently for all electronic states arising from this definition of the active space. The spin–orbit interaction (described within the AMFI approximation) includes all optimized spin states for Er (**3** and **4**), while for **2** we could only mix a limited amount of states, namely 21 spin sextet, 128 spin quartet and 130 spin doublet states, which resulted in 898 spin–orbit levels. The obtained low-lying states, arising from the ground *J* = 15/2 multiplet on individual Ln^III^ sites, are provided in Table 1.

#### Structural features determining the orientation of local magnetic axes on Ln^III^ sites

**Fig. 5:**
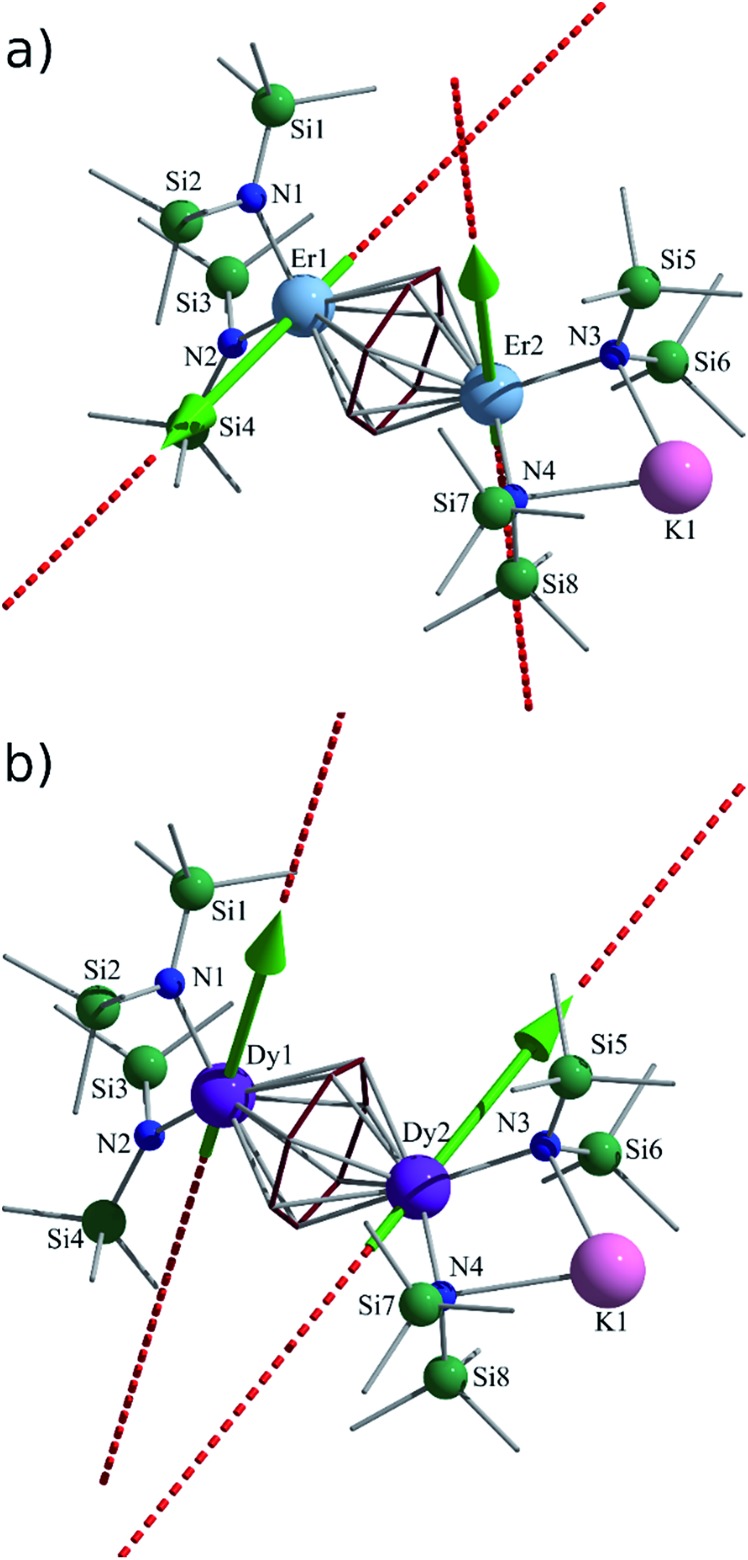
*Ab initio* calculated main anisotropy axes (dashed lines) on Ln sites in the ground state for (a) **3** and (b) **2**. The green arrows show the orientation of local magnetic moments on Ln sites in the ground exchange coupled state.

As can be observed in Table 1, the *g* tensors in the ground Kramers doublet states of the individual sites in compounds **2–4** are relatively axial in nature (*g*_*X*,*Y*_ ≪ *g*_*Z*_). The axiality of the ground doublet states are also related to the axiality of the crystal field acting on the Ln^III^ sites. For the Dy^III^ sites, the main anisotropy axis is oriented in the plane of the N–Dy–N atoms (Table 1) almost parallel to the N–N direction (Fig. 5b). This orientation is related to the much stronger crystal field effect arising from the N atoms. In particular, the calculated LoProp charges^56^ on N atoms (–1.28) are the largest among all neighbouring atoms of the Ln^III^ sites. The covalent ligand field effect arising from the N atoms is also dominant among all neighbouring atoms. This is revealed by the Dy–N bonds, which are the shortest formed by the lanthanide sites in this environment. In this respect, the role of the central ring in the local axiality of the Dy^III^ sites is diminished, and is in fact rather destructive as compared to the ligand field imposed by amido groups. In the case where the central ring and the neighbouring Ln^III^ site were absent, the magnetic axiality on one Dy^III^ site would be significantly stronger. These findings were not surprising given that recent reports have demonstrated the significant impact of highly anionic donor ligands in linear-like coordination geometries, with which such compounds should theoretically yield staggering energy barrier values.^44–47^

In stark contrast to the findings for Dy^III^, significantly different orientations for the ground state magnetic anisotropies were observed for the Er^III^ sites in compounds **3** and **4** (Table 1, Fig. 5a). For these compounds, the main magnetic axes are oriented almost perpendicular to the N–Er–N planes. This drastic change in the orientations of the main magnetic axes between Dy^III^ and Er^III^ atoms in a very similar axial ligand field is due to the opposite signs of the Stevens parameters, *α* and *β*, which are related to the second and fourth rank operators of the ground ionic *J* = 15/2 multiplet for Dy^III^ and Er^III^.^57^ This is seen from the fact that the anisotropy of the highest (8^th^) Kramers doublet of the Dy^III^ sites (which are greatly destabilized due to the crystal field) is in fact almost parallel to the anisotropy of the ground doublet for the Er^III^ ion in the same crystal field, thus demonstrating the complementary nature of Dy^III^ and Er^III^ ions. This effect was previously observed in the case of [Er(COT)_2_]^–^ and [Dy(COT)_2_]^–^ anions.^42^ Similar arrangements of the local magnetic axes were revealed with the previously studied Er_2_C_6_ (η^6^-C_6_R_6_) compounds.^22^ Parameters of the *ab initio* calculated crystal field for the investigated Ln sites in **2–4** are given in Table S2.†

#### Exchange interaction in **2–4**

The above reported *ab initio* results for separate Ln^III^ sites in **2–4** were further employed in the computation of the exchange spectrum and magnetic properties of the dinuclear complexes using the POLY_ANISO program.^58,59^ In this approach, the exchange interaction between magnetic sites is considered within the Lines model,^60^ describing the exchange interaction between the localized spins in the absence of the spin–orbit interaction on sites by one parameter for the interacting metal pair. By explicitly considering the spin–orbit interaction on metal sites, the Lines model leads to an exchange matrix, which effectively describes the anisotropic exchange interaction between sites. In particular, the contribution of the intramolecular dipole–dipole magnetic coupling is accounted for exactly, because all of the necessary data are made available through the *ab initio* calculations. On the basis of the resulting exchange spectrum of the entire system, all macroscopic magnetic properties were computed. The total magnetic interaction (exchange + dipolar) between the lowest Kramers doublets on lanthanide sites can be cast in a good approximation by the non-collinear Ising Hamiltonian:

1 *Ĥ*_exch_ = –(*J*_exch_ + *J*_dip_)*Ŝ*_1z_*Ŝ*_2z_

where *J*_exch_ and *J*_dip_ are parameters of the exchange and dipolar couplings respectively, while *Ŝ*_1z_ = 1/2 is the pseudospin of the ground states of the metal sites. Best-fit exchange parameters, *J*_exch_, and the calculated parameters of the dipolar magnetic coupling, *J*_dip_, for the investigated compounds, alongside the resulting exchange spectra are given in Table 2.

An alternative approach for the estimation of the exchange coupling parameters in di- and poly-nuclear compounds is given by the broken-symmetry density functional theory approach (BS-DFT).^61^ Unfortunately, the BS-DFT approach is not directly applicable for most of the lanthanides given the multiconfigurational nature of their ground states and their near-degenerate status as a result of weak crystal field effects. However, an estimation of the exchange in lanthanide-containing compounds is still achievable from the BS-DFT calculations. To this end, the “isotropic” closest metals computationally replace the “anisotropic” metal sites of the investigated compounds, while the ligand framework is kept intact. BS-DFT calculations are performed straightforwardly for the “isotropic” equivalent of the investigated compound. The extracted *J*_iso_ parameter has to be later rescaled to reflect the exchange Hamiltonian between the true spins of the original “anisotropic” metal sites. This method was employed with reasonable success in several previous studies.^63^ For the present compounds, the estimated exchange parameters from the BS-DFT studies are ferromagnetic 1.14 cm^–1^ for **2**, 2.45 cm^–1^ for **3** and 3.16 cm^–1^ for **4**, correlating reasonably with the ferromagnetic exchange values obtained within the Lines model (Table 2). A comparison between the calculated and measured magnetic susceptibilities is depicted in Fig. 2. We notice a clear reduction in the dipolar magnetic coupling values for **2–4** with respect to our previously investigated dinuclear compounds containing a 6-membered bridging moiety.^22^ The reduction of *J*_dip_ is attributed to the different relative orientations of the local magnetic axes of the two Ln^III^ sites, imposed by the different dihedral angles between the N–Ln–N planes. Thus, by controlling this angle through synthetic means we could, in principle, modify the magnetic dipolar interaction (and possibly the exchange) in such compounds. Through this study, we attempted to computationally assess the role of the dihedral angle between N–Ln–N planes in the dinuclear model systems **2–4**, as well as the role of the bridging ligand, in the magnetic behaviour as compared to those with 6- and 8-membered bridging rings. The results show that the dihedral angle in all three cases is very similar, while the Ln–Ln distance decreases with increasing bridging ring size. We conclude, therefore, that the bulky ancillary ligands (*i.e.* [N(SiMe_3_)_2_]^–^ ligands), and the resulting crystalline packing, are the factors responsible for defining the relative orientations of the local anisotropy axes and dipolar magnetic interaction in this series of compounds.

## Conclusions

Compounds **1–4** represent the first examples of SMMs based on the cycloheptatrienyl trianion ligand. The synthetic route to achieve the aforementioned compounds has been carefully designed to yield the facile formation of the trianion, taking advantage of sterically demanding and highly basic ancillary ligands. When combined with lanthanide ions, this type of bridging motif generates a weak, yet non-negligible, magnetic coupling constant of *J* = –0.134 cm^–1^ for the isotropic analogue. Through computational modelling of the anisotropic compounds, we elucidated that exchange coupling is more significant than dipolar coupling, with the largest *J*_exch_ being +3.149 cm^–1^ for compound **4**, thereby demonstrating the desirable effects of the 7-membered bridging moiety in generating exchange coupled dinuclear lanthanide systems. This is an area of significant modern interest in quantum physics, where mediating the interaction of two metal centres *via* tuning the redox properties of the bridging motif is a method to induce significant quantum communication.^64,65^ Moreover, the incorporation and measurement of these materials in molecular spintronics devices are often limited to the millikelvin regime,^66^ where the surface effects of such materials is only beginning to be better understood.^62^ Hence, increasing the energy barrier to spin reversal of SMMs will relax the rigorous experimental requirements for studying these systems. Thus, the current high-energy barriers associated with 4f ions, attributed to single-ion behaviour, will not be sufficient. It is vital that we look for more creative ways to induce significant interactions between lanthanide ions.
