# Supplementary material for: Cycloheptatrienyl trianion: an elusive bridge in the search of exchange coupled dinuclear organolanthanide single-molecule magnets
Source: Chem Sci. 2016 Aug 8;8(1):231–40. doi: 10.1039/c6sc01224h (PMC5355948; doi:10.1039/c6sc01224h)
Supplement: Supplementary file 1 [file SC-008-C6SC01224H-s001.pdf]

Supporting Information

Cycloheptatrienyl trianion: An elusive bridge in the search of exchange coupled dinuclear organolanthanide single-molecule magnets

Katie L. M. Harriman,[a]† Jennifer J. Le Roy,[a]† Liviu Ungur,[,b,c] Rebecca J. Holmberg,[a] Ilia Korobkov,[a] and Muralee Murugesu*[,a]*

[a] Department of Chemistry and Biomolecular Sciences and Centre for Catalysis Research and Innovation, University of Ottawa, Ottawa, Ontario K1N 6N5, Canada.
[b] Theory of Nanomaterials Group and INPAC – Institute of Nanoscale Physics and Chemistry, Katholieke, Universiteit Leuven, Celestijnenlaan 200F, 3001 Leuven Belgium.
[c] Theoretical Chemistry, Lund University, Getingevagen 60, 22241, Lund Sweden.

† Both authors contributed equally to this work

*Corresponding Author: M. Murugesu (E-mail: m.murugesu@uottawa.ca; Tel: +1 (613) 562 5800 ext 2733)

Contents

Page S2 Experimental Section
Page S5 X-Ray Powder Diffraction
Page S7 Single Crystal X-Ray Diffraction
Page S11 Magnetic Measurements
Page S17 Results of *Ab initio* Investigation
Page S23 References

General Procedures and Starting Materials. All reactions and subsequent manipulations were performed under anaerobic and anhydrous conditions using a standard Schlenk-line or nitrogen-atmosphere glovebox. Tetrahydrofuran (THF), toluene, and n-hexanes were dried on columns of activated alumina using a J.C. Mayer solvent purification system. Pentanes was dried *via* distillation over calcium hydride and subsequent passage over activated alumina. All solvents were of least reagent grade and were stored over 3Å molecular sieves. Anhydrous toluene-d_8 and anhydrous chloroform-d was purchased from CDN Isotopes and Sigma Aldrich respectfully, and used without further purification. Anhydrous $LnCl_3$ was purchased from Strem Chemicals, sodium *bis*(trimethylsilyl)amide, potassium, and 1,3-cycloheptadiene was purchased from Sigma Aldrich; these chemicals were used as received. Triethylamine was purchased from Alfa Aesar and dried by refluxing over calcium hydride, distillation, and storage over 3Å sieves. Potassium cycloheptadienide[1] and lanthanide *tris*(bis(trimethylsilylamido))[2] were prepared from previously reported procedures with modifications as described below. Infrared spectra were recorded on a Nicolet Nexus 550 FT-IR spectrometer using transmission mode in the 4000-600 cm^{-1} range; solid samples were prepared under an inert atmosphere and sandwiched between transparent NaCl plates. Elemental analyses were carried out with a Costech EOS 4010 CHNSO analyzer. NMR spectra were acquired on a Bruker Avance-II 300 MHz spectrometer at 298 K

Preparation of K(C_7H_9). Under an inert atmosphere, K^0 (360 mg, 9.22 mmol) was added to a reaction vessel containing 6 mL of THF and 0.96 mL of Et_3N (6.91 mmol). With vigorous stirring 1,3-cycloheptadiene (2 mL, 18.4 mmol) was added slowly to the vessel at 0 ^oC. Following the addition of the diene, the vessel was allowed to gradually warm to room temperature for 16 h. Addition of n-hexanes and subsequent exposure to -35 ^oC yielded yellow needle-like crystals. Collection of the crystals over a fine frit and subsequent washings with n-hexanes provided the product in 70% yield. IR (neat, cm^{-1}): 2826 (w), 1557 (m), 1529 (m), 1444 (m), 1363 (m), 1318 (m), 1274 (w), 1235 (w), 1215 (w), 1155 (m), 1125 (m), 1041 (m), 1006 (w), 948 (w), 889 (s), 867 (s), 771 (w), 710 (s), 634 (w), 541 (s). ^1H NMR ($CDCl_3$, 298 K, δ/ppm): 5.79 (m, 5H, C_{sp3}-*H*), 2.13 (m, 4H, C_{sp2}-*H*).

Preparation of [Ln(N($SiMe_3$)$_2$)$_3$], Ln = Gd, Dy, Er. Under an inert atmosphere, a slurry of $LnCl_3$ (1.86 mmol) in THF was combined with a THF solution of NaN($SiMe_3$)$_2$ (5.57 mmol) at -35 ^oC. The reaction mixture was gradually warmed to room temperature over 16 h. Solvent was removed

under reduced pressure and the subsequent solid treated with n-pentanes. The solution was filtered over Celite, the collection of the filtrate and storage at -35 °C, resulted in the formation of needle-like crystals of $[Ln(N(SiMe_3)_2)_3]$. Unit cell determinations matched previously reported structures for Ln = Dy and Er, however; no previous report of Ln = Gd exists.

Select characterizations for $[Gd(N(SiMe_3)_2)_3]$. Yield = 52 % IR (neat, cm^{-1}): 2934 (br), 1436 (m), 1235 (s), 1186 (w), 1094 (w), 1045 (w), 978 (s), 807 (w), 765 (w), 655 (m), 601 (s). ^1H NMR ($CDCl_3$, 298 K, δ/ppm): -11.67 (br, s, $Si(CH_3)_3$, FWHM: 5233.11 Hz).

Select characterizations for $[Dy(N(SiMe_3)_2)_3]$. Yield = 49 % IR (neat, cm^{-1}): 2944 (br), 1424 (m), 1236 (s), 1090 (w), 1048 (w), 974 (s), 827 (m), 768 (m), 656 (s), 604 (s). ^1H NMR ($CDCl_3$, 298 K, δ/ppm): -102.37 (br, s, $Si(CH_3)_3$, FWHM: 846.39 Hz).

Select characterizations for $[Er(N(SiMe_3)_2)_3]$. Yield = 51 % IR (neat, cm^{-1}): 2945 (br), 1423 (m), 1235 (s), 1090 (w), 1050 (w), 972 (s), 827 (m), 769 (m) 654 (m), 603 (m). ^1H NMR ($CDCl_3$, 298 K, δ/ppm): 62.86 (br, s, $Si(CH_3)_3$, FWHM: 2670.44 Hz).

Preparation of $[KGd_2(C_7H_7)(N(SiMe_3)_2)_4]$ (1). Under an inert atmosphere, $Gd(N(SiMe_3)_2)_3$ (58%, 0.350 mmol) was added to a cold solution of $K(C_7H_9)$ (80 mg, 0.605 mmol) at -35 °C in 10 mL of toluene. The reaction mixture was stirred for 12 h, gradually warming to room temperature. The filtrate was then collected and solvent was removed under reduced pressure to achieve an orange-brown oil. Subsequent treatment with toluene and n-hexanes yielded X-ray quality crystals of **1**, (yield = 22 - 26 %). Isolated X-ray diffraction quality crystals are air and moisture sensitive. Bulk purity of compound **1** was determined by X-ray powder diffraction, *vide infra*. IR (neat, cm^{-1}): 2940 (w), 2892 (w), 1587 (br), 1494 (w), 1433 (br), 1240 (s), 1180 (w), 1066 (w), 992 (m), 931 (w), 872 (m), 815 (s), 740 (m), 693 (w), 655 (m). ^1H NMR (toluene-d_8, 298 K, δ/ppm): -196.04 (br, s, 17.8H, $Si(CH_3)_3$, FWHM: 24.52 Hz), -196.93 (br, s, 7H, C_7H_7, FWHM: 34.76 Hz), -197.28 (br, s, 16.9H, $Si(CH_3)_3$, FWHM: 34.46 Hz), -198.09 (br, s, 35.8H, $Si(CH_3)_3$, FWHM: 26.38 Hz). ^1H NMR integrations have been calibrated to seven protons on C_7H_7. Anal. Calcd. for $C_{31}H_{79}Gd_2KN_4Si_8$: C, 34.28; H, 7.33; N, 5.16. Found: C, 34.60; H, 7.12; N, 4.93.

Preparation of $[KDy_2(C_7H_7)(N(SiMe_3)_2)_4]$ (2). Compound **2** was prepared in an analogous manner to **1.** X-ray quality crystals of **2**, (yield = 22 - 26 %). Isolated X-ray diffraction quality crystals are air and moisture sensitive. Bulk purity of compound **2** was determined by X-ray

powder diffraction, *vide infra*. IR (neat, cm^{-1}): 2939 (w), 2891 (w), 1595 (br), 1494 (w), 1421 (br), 1241 (s), 1180 (w), 1083 (w), 999 (m), 932 (w), 879 (m), 820 (s), 729 (m), 694 (w), 662 (m). ^1H NMR (toluene-d_8, 298 K, δ/ppm): 95.43 (br, s, 17.5H, $Si(CH_3)_3$, FWHM: 3604.57 Hz), -34.12 (br, s, 17.9H, $Si(CH_3)_3$, FWHM: 3739.31 Hz), -76.68 (br, s, 36.9H, $Si(CH_3)_3$, FWHM: 305.09 Hz), -100.81 (br, s, 7H, C_7H_7, FWHM: 256.67 Hz). ^1H NMR integrations have been calibrated to seven protons on C_7H_7. Anal. Calcd. for $C_{31}H_{79}Dy_2KN_4Si_8$: C, 33.95; H, 7.26; N, 5.11. Found: C, 33.71; H, 6.97; N, 5.43.

Preparation of [$KEr_2(C_7H_7)(N(SiMe_3)_2)_4$] (3). Compound **3** was prepared in an analogous manner to **1.** X-ray quality crystals of **3**, (yield = 22 - 26 %). Isolated X-ray diffraction quality crystals are air and moisture sensitive. Bulk purity of compound **3** was determined by X-ray powder diffraction, *vide infra*. IR (neat, cm^{-1}): 2949 (w), 2892 (w) 1662 (w), 1603 (w), 1495 (w), 1435 (br), 1249 (s), 1180 (m), 1079 (w), 988 (m), 931 (m), 882 (w), 822 (s), 728 (m), 693 (w), 661 (m). ^1H NMR (toluene-d_8, 298 K, δ/ppm): 45.98 (br, s, 17.6H, $Si(CH_3)_3$, FWHM: 723.42 Hz), 40.81 (br, s, 37.2H, $Si(CH_3)_3$, FWHM: 758.32 Hz), 15.85 (br, s, 7H, C_7H_7, FWHM: 1807.98 Hz), -98.44 (br, s, 17.8H, $Si(CH_3)_3$, FWHM: 963.65 Hz). ^1H NMR integrations have been calibrated to seven protons on C_7H_7. Anal. Calcd. for $C_{31}H_{79}Er_2KN_4Si_8$: C, 33.65; H, 7.20; N, 5.06. Found: C, 33.26; H, 7.09; N, 4.86.

Preparation of [$K(THF)_2Er_2(C_7H_7)(N(SiMe_3)_2)_4$] (4). Under an inert atmosphere, $Er(N(SiMe_3)_2)_3$(223 mg, 0.350 mmol) was added to a cold solution of $K(C_7H_9)$ (80 mg, 0.605 mmol) at -35 °C in 10 mL of toluene. The reaction mixture was stirred for 12 h, gradually warming to room temperature. The filtrate was collected, extracted into THF, and the subsequent filtrate was evaporated to dryness with reduced pressure to achieve an orange-brown oil. Treatment with toluene and n-hexanes yielded x-ray quality crystals of **4**, (yield = 32 %). Isolated X-ray diffraction quality crystals are air and moisture sensitive. IR (neat, cm^{-1}): 2941 (s), 2884 (s), 1461 (w), 1425 (m), 1369 (w), 1243 (s), 1181 (w), 1085 (s), 1052 (m), 989 (m), 869 (m), 818 (m), 753 (m), 711 (w), 691 (w), 662 (m), 603 (m). ^1H NMR (toluene-d_8, 298 K, δ/ppm): 45.15 (br, s, 17.9H, $Si(CH_3)_3$, FWHM: 914.35 Hz), 40.86 (br, s, 35.7H, $Si(CH_3)_3$, FWHM: 883.96 Hz), 35.62 (br, s, 7.9H, THF, FWHM: 570.66 Hz), 21.36 (br, s, 7.9H, THF, FWHM: 594.09 Hz), 15.60 (br, s, 7H, C_7H_7, FWHM: 110.97 Hz), -99.35 (br, s, 17.7H, $Si(CH_3)_3$, FWHM: 184.19 Hz). ^1H NMR

integrations have been calibrated to seven protons on C_7H_7. Anal. Calcd. for $C_{39}H_{95}Er_2KN_4O_2Si_8$: C, 37.46; H, 7.66; N, 4.48. Found: C, 37.91; H, 7.29; N, 4.51.

X-ray Powder Diffraction (XRPD). XRPD experiments were performed using a RIGAKU Ultima IV diffractometer, equipped with a Cu-Kα radiation source (λ = 1.541836 Å), and a graphite monochromator. Scanning of the 2θ range was performed from 5-35°. XRPD pattern was consistent in 2θ values with the generated pattern from XRD, with slight discrepancies in some intensities of peaks attributed to preferred orientation and some broad amorphous character present.

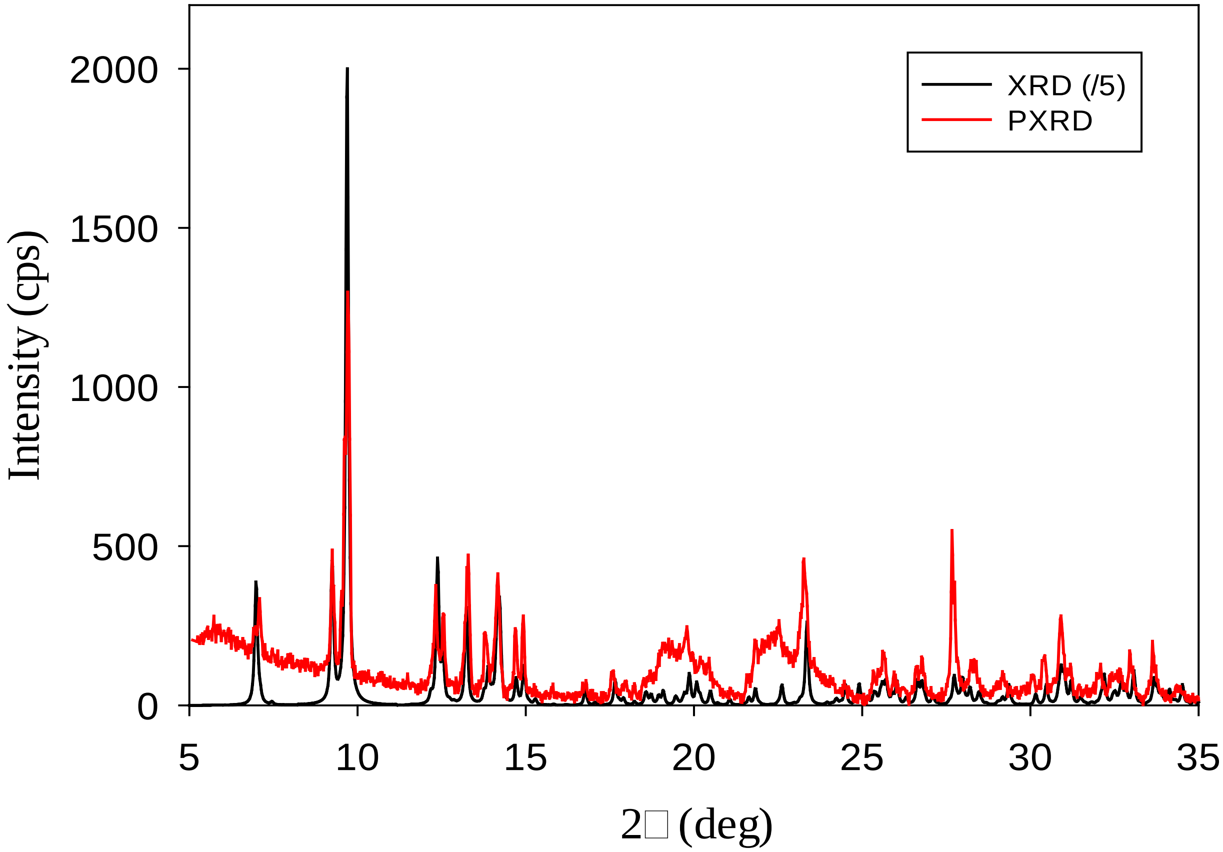

Figure S1. X-ray powder diffraction of **[KGd$_2$(C$_7$H$_7$)(N(SiMe$_3$)$_2$)$_4$]**, **1**, (red) in the 5-35° 2θ region, as compared with the theoretical pattern generated from single crystal X-ray data (black).

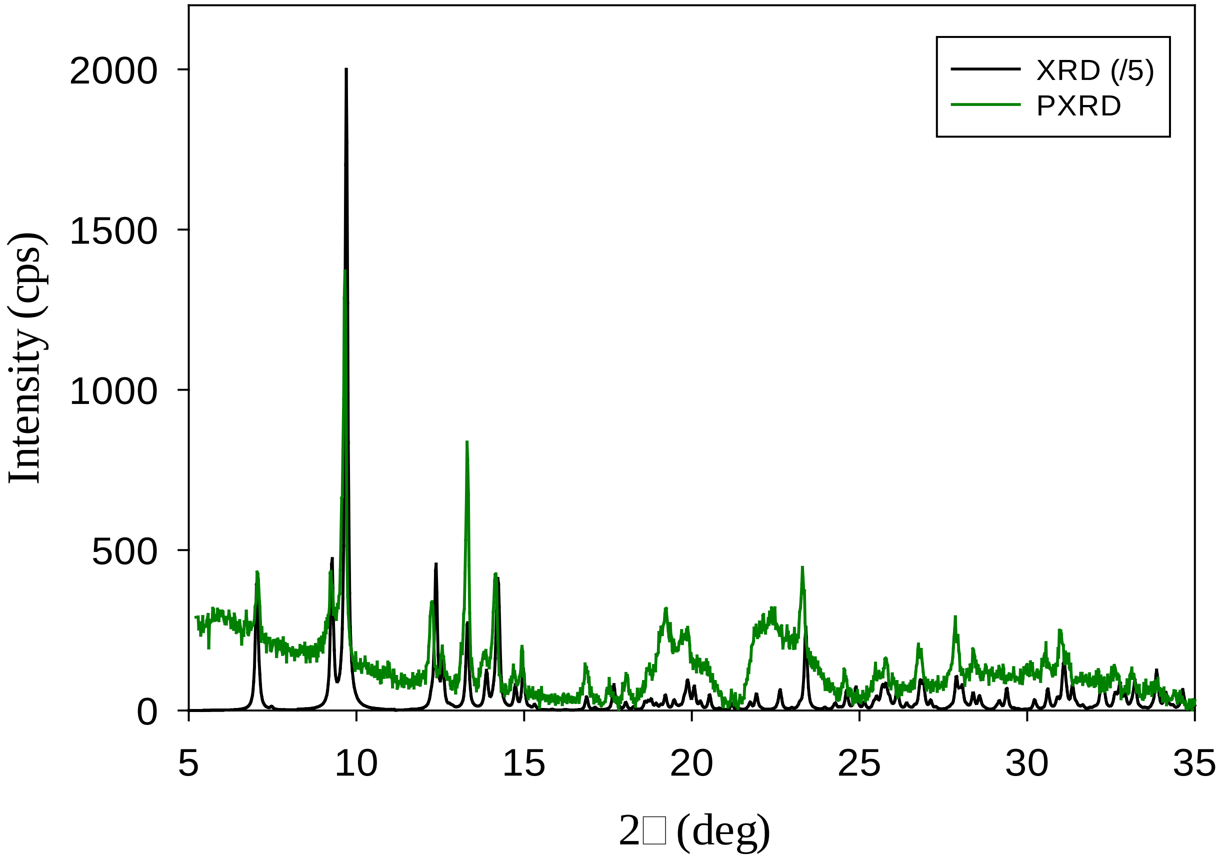

Figure S2. X-ray powder diffraction of **[$KDy_2(C_7H_7)(N(SiMe_3)_2)_4$]**, **2**, (green) in the 5-35° 2θ region, as compared with the theoretical pattern generated from single crystal X-ray data (black).

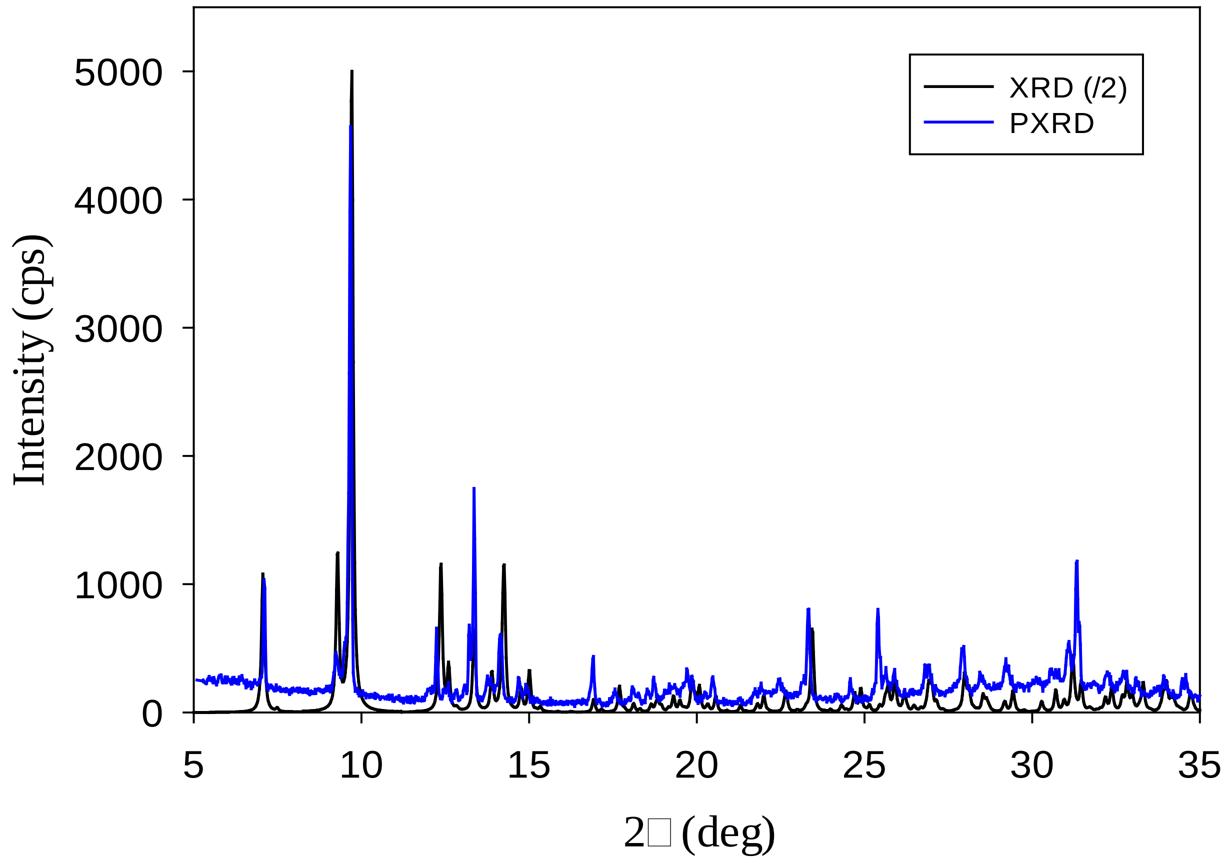

Figure S3. X-ray powder diffraction of **[$KEr_2(C_7H_7)(N(SiMe_3)_2)_4$]**, **3**, (blue) in the 5-35° 2θ region, as compared with the theoretical pattern generated from single crystal X-ray data (black).

Single Crystal X-ray Diffraction Studies. Data collection results for compounds represent the best data sets obtained in several trials for each sample. The crystals were mounted on thin glass fibers using paraffin oil. Prior to data collection crystals were cooled to 200.15 K. Data were collected on a Bruker AXS KAPPA single crystal diffractometer equipped with a sealed Mo tube source (wavelength 0.71073 Å) APEX II CCD detector. Raw data collection and processing were performed with APEX II software package from BRUKER AXS.[3] Diffraction data was collected with a sequence of 0.30° ω scans at 0.00, 120.0, and 240.0° in φ. Initial unit cell parameters were determined from 60 data frames with 0.3° ω scan each, collected at the different sections of the

Ewald sphere. Semi-empirical absorption corrections based on equivalent reflections were applied.[4] Systematic absences in the diffraction data-set and unit-cell parameters were consistent with monoclinic **P***2₁/n* (№11) for compounds **1-3**, and monoclinic **C***2/c* (№15) for compound **4**. Solutions in the centrosymmetric space groups for all samples yielded chemically reasonable and computationally stable results of refinement. Structures were solved by direct methods, completed with difference Fourier synthesis, and refined with full-matrix least-squares procedures based on F^2.

For all the compounds all hydrogen atoms positions were calculated based on the geometry of related non-hydrogen atoms. All hydrogen atoms were treated as idealized contributions during the refinement. All scattering factors are contained in several versions of the SHELXTL program library, with the latest version used being v.6.12.[5] Crystallographic data and selected data collection parameters are reported in Table S1.

Table S1. Crystallographic data and selected data collection parameters for dinuclear compounds **1-4**.

**Compound**	**1**	**2**	**3**	**4**
Empirical formula	$C_{31}H_{79}Gd_2KN_4Si_8$	$C_{31}H_{79}Dy_2KN_4Si_8$	$C_{31}H_{79}Er_2KN_4Si_8$	$C_{39}H_{95}Er_2KN_4O_2Si_8$
Formula weight	1086.30	1096.80	1106.32	1250.52
Crystal size, mm	0.290x0.203x0.139	0.210x0.140x0.120	0.800x0.543x0.430	0.677x0.530x0.200
Crystal system	Monoclinic	Monoclinic	Monoclinic	Monoclinic
Space group	**P***2₁/n*	**P***2₁/n*	**P***2₁/n*	**C***2/c*
Z	4	4	4	4
a, Å	14.1700(7)	14.1259(4)	14.09464(7)	20.1042(7)
b, Å	14.2848(7)	14.2954(4)	14.2965(8)	18.7574(6)
c, Å	25.4039(12)	25.2343(6)	25.1055(13)	16.5696(6)
α, °	90	90	90	90
β, °	95.518(2)	95.2741(13)	95.170(3)	107.8505(17)
γ, °	90	90	90	90
Volume, Å^3	5118.3(4)	5074.1(2)	5038.3(5)	5947.6(4)
Calculated density, Mg/m^3	1.410	1.436	1.459	1.397

Absorption coefficient, mm^{-1}	2.861	3.217	3.606	3.066
T (K)	200(2)	200(2)	200(2)	200(2)
F(000)	2208	2224	2240	2560
Θ range for data collection, °	1.584 to 30.595	1.723 to 28.424	1.598 to 30.659	1.520 to 28.381
Limiting indices	h = ±19, k = ±20, l = ±36	h = ±18, k = ±18, l = ±33	h = ±20, k = ±20, l = ±35	h = ±26, k = ±25, l = ±22
Reflections collected / unique	25502	58488	26202	23004
R(int)	-	0.0334	-	0.0258
Completeness to Θ = 28.32, %	99.6	99.3	99.5	98.1
Max. and min. transmission	0.746071 and 0.531793	0.7457 and 0.5770	0.746065 and 0.304526	0.7457 and 0.4771
Data / restraints / parameters	15195 / 0 / 416	12645 / 0 / 415	15164 / 0 / 416	7321 / 657 / 286
Goodness-of-fit on F^2	1.055	1.019	1.063	1.071
Final R indices [I>2σ(I)]	R1 = 0.0330, wR2 = 0.0626	R1 = 0.0299, wR2 = 0.0642	R1 = 0.0443, wR2 = 0.1120	R1 = 0.0384, wR2 = 0.1251
R indices (all data)	R1 = 0.0544, wR2 = 0.0725	R1 = 0.0424, wR2 = 0.0693	R1 = 0.0648, wR2 = 0.1336	R1 = 0.0438, wR2 = 0.1325
Largest diff. peak/hole, $e \cdot Å^{-3}$	2.022 and -0.913	1.430 and -1.064	3.653 and -2.021	1.380 and -2.107

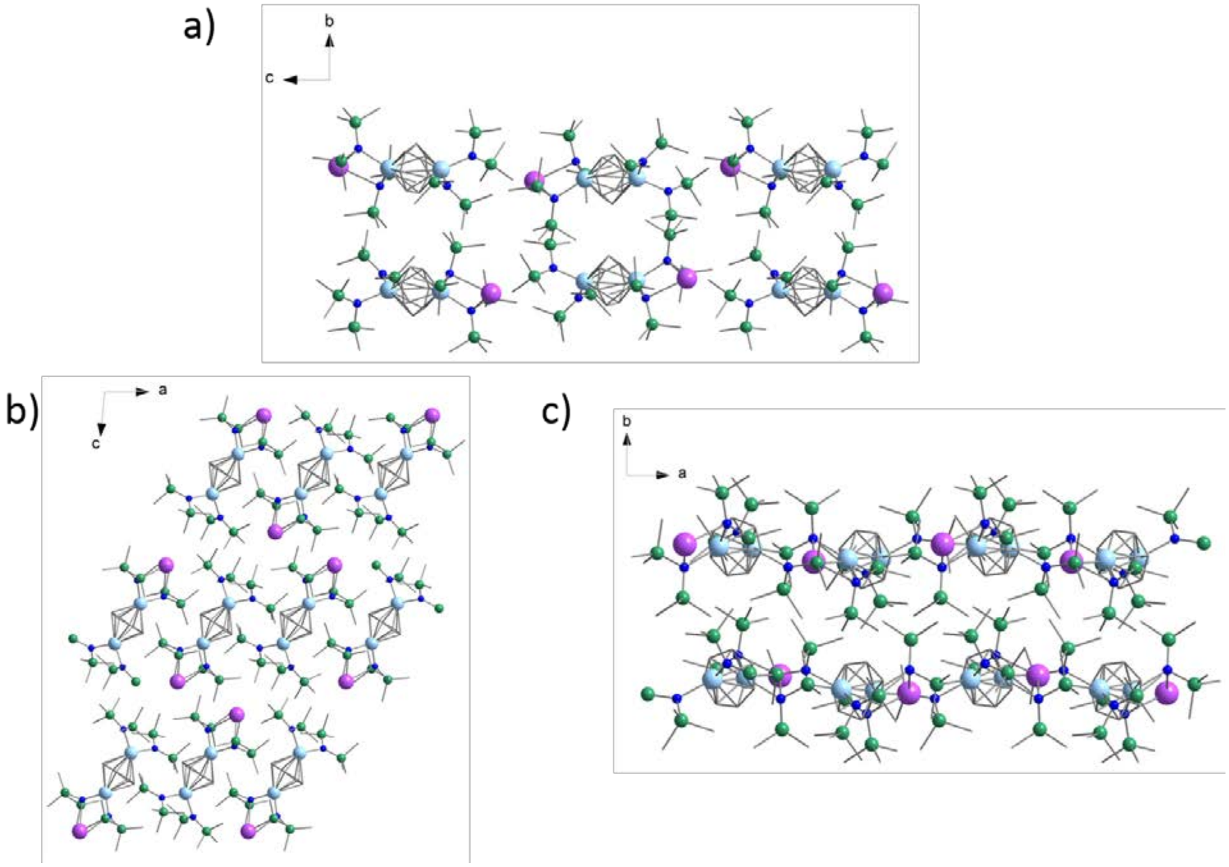

Figure S4. Crystallographic packing diagrams of **[$KEr_2(C_7H_7)(N(SiMe_3)_2)_4$] (3),** (a) along the *a*-axis, (b) along the *b*-axis, and (c) along the *c*-axis. Isostructural compounds **1** and **2** exhibit analogous packing arrangements. Colour code: Er; light blue, Si; green, K; purple, N; royal blue, C; grey.

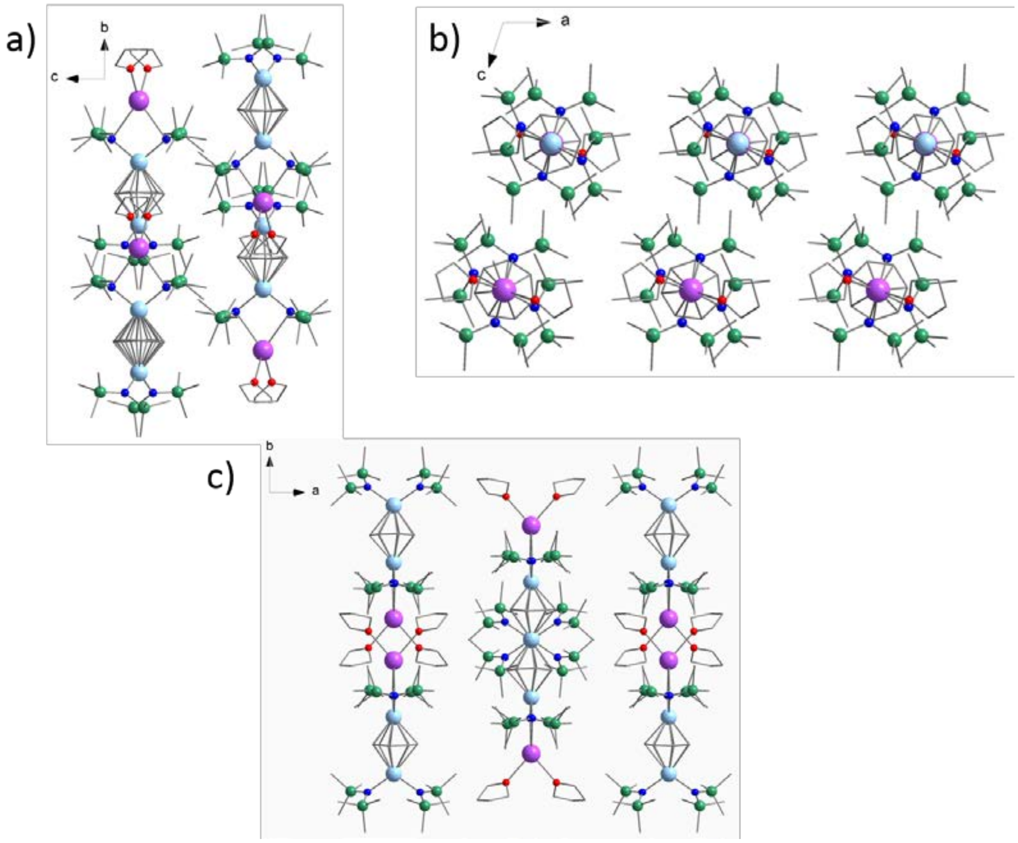

Figure S5. Crystallographic packing diagrams of **[K(THF)$_2$Er$_2$(C$_7$H$_7$)(N(SiMe$_3$)$_2$)$_4$] (4)** (a) along the *a*-axis, (b) along the *b*-axis, and (c) along the *c*-axis. Colour code: Er; light blue, Si; green, K; purple, N; royal blue, O; red, C; grey.

Magnetic Measurements. The magnetic susceptibility measurements were obtained using a Quantum Design SQUID magnetometer MPMS-XL7 operating between 1.8 and 300 K. DC measurements were performed on a polycrystalline samples of 12 mg, 15 mg, 11 mg, and 21 mg of **1-4** respectively. Samples were wrapped in a polyethylene bag, and subjected to a field of 0 to 7 T. The magnetization data were collected at 100 K to check for ferromagnetic impurities that were absent in all of the samples. Diamagnetic corrections were applied for the sample holder and the inherent diamagnetism of the sample was estimated with the use of Pascals constants.

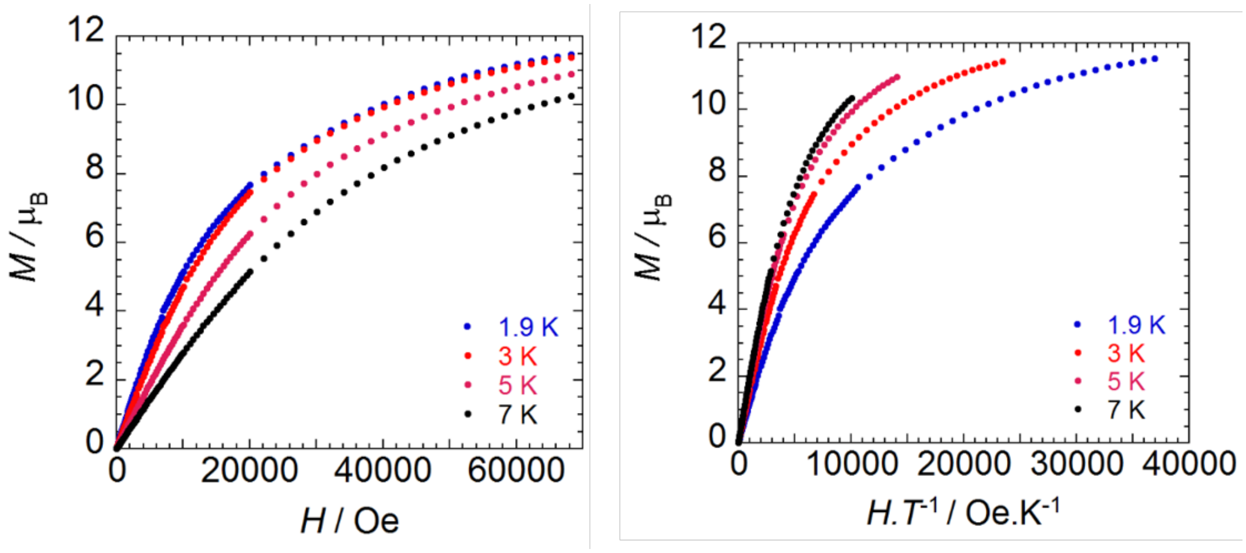

Figure S6. Solid state field dependence of the magnetization (left) and reduced magnetization (right) for **1** at the indicated temperatures.

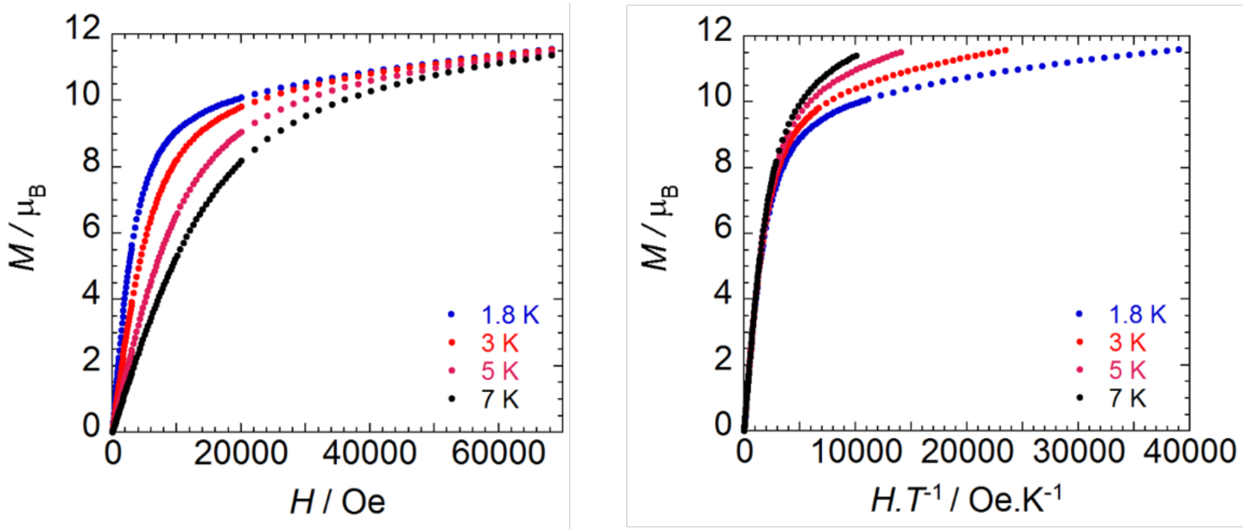

Figure S7. Solid state field dependence of the magnetization (left) and reduced magnetization (right) for **2** at the indicated temperatures.

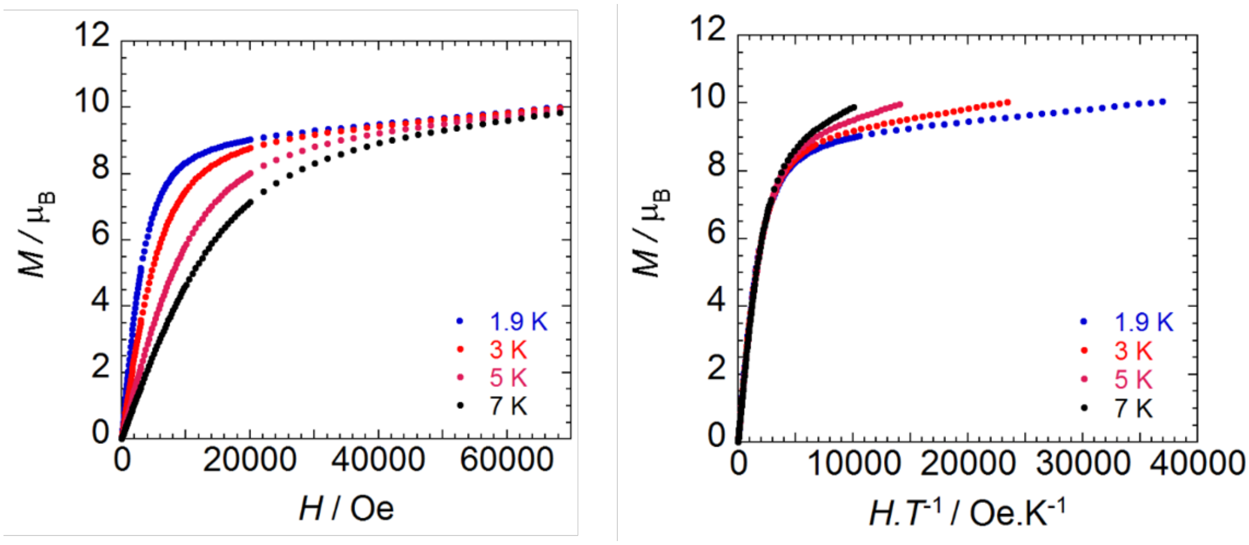

Figure S8. Solid state field dependence of the magnetization (left) and reduced magnetization (right) for **3** at the indicated temperatures.

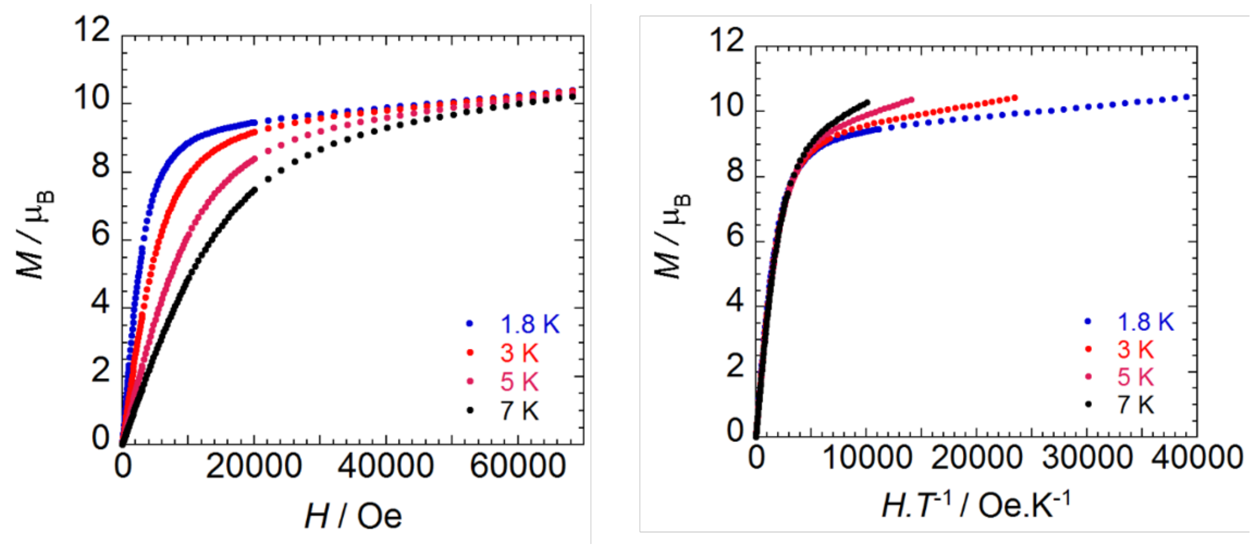

Figure S9. Solid state field dependence of the magnetization (left) and reduced magnetization (right) for **4** at the indicated temperatures.

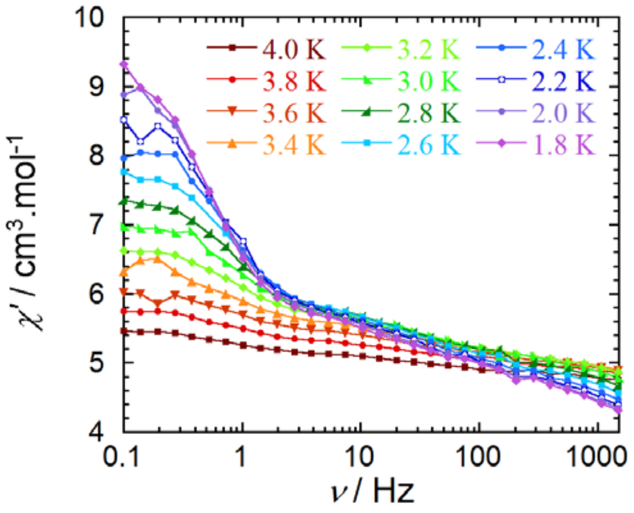

Figure S10. Variable temperature in-phase (χ') magnetic susceptibility of **2** under a static dc field of 2000 Oe at the indicated temperatures.

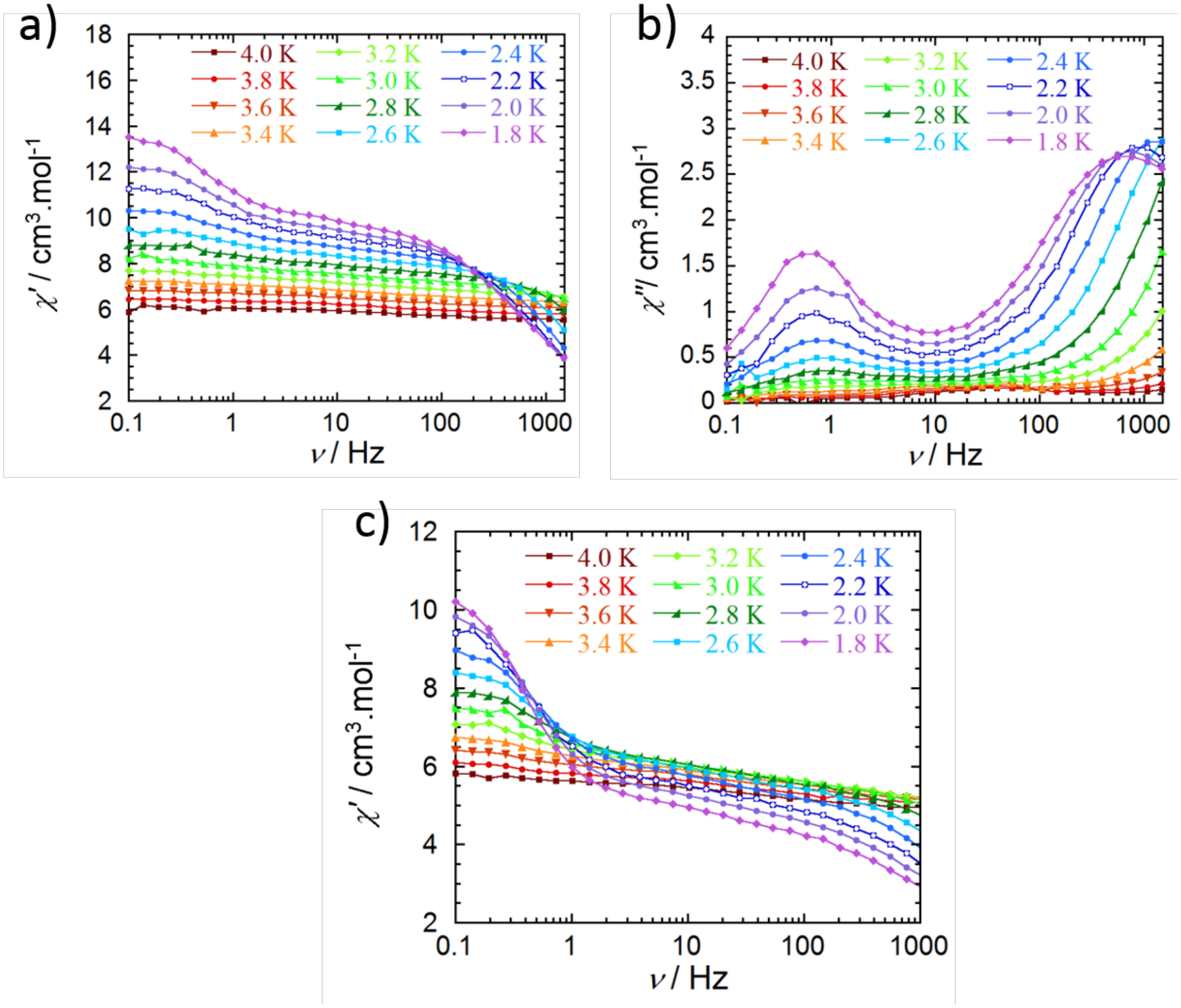

Figure S11. Variable temperature in-phase (χ') (left) and out-of-phase (χ'') (right) magnetic susceptibility of **4** under a static dc field of 1000 Oe for a) and b), and a static dc field of 2000 Oe for c).

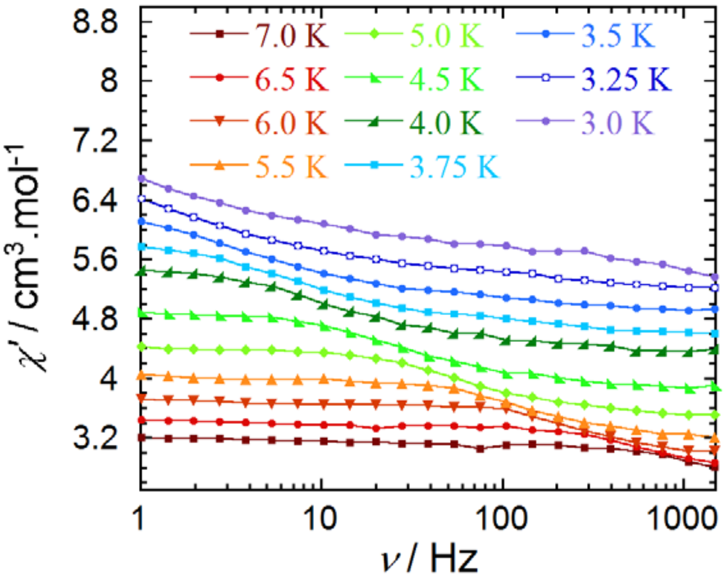

Figure S12. Variable temperature in-phase (χ') magnetic susceptibility of **3** under a static dc field of 800 Oe at the indicated temperatures.

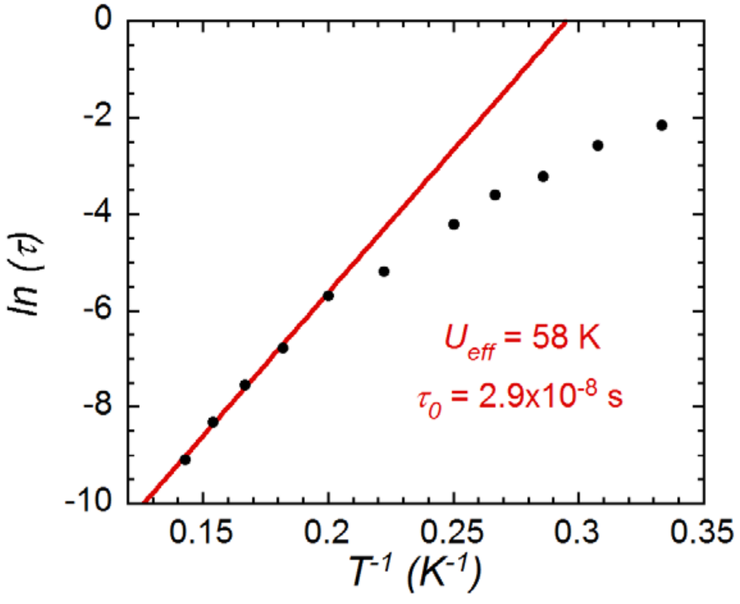

Figure S13. Plot of ln(τ) versus T for **3** at H_{dc} = 800 Oe. The solid lines represent the Arrhenius fit of the frequency-dependent data.

Supplementary details and results of the *ab initio* investigation

All atoms were described with ANO-RCC basis sets.
MB- basis = minimal
VDZP-basis = medium
VTZP-basis = large
Active space of the CASSCF method included the $4f^n$ configuration only. Active orbitals have a weight of ~95% on the atomic 4*f* basis of the lanthanide.
For Dy sites in 2, 21 spin sextet, 128 spin quartet and 130 spin doublet states were mixed by the spin-orbit coupling within RASSI method.

Table S2: Energies of the spin-free states (CASSCF, cm^{-1}) obtained (cm^{-1}) for compound 2

Spin	center Dy1			center Dy2 (closer to K)		
	MB	VDZP	VTZP	MB	VDZP	VTZP
5/2	0.000	0.000	0.000	0.000	0.000	0.000
	2.474	4.246	3.178	3.137	4.691	1.722
	409.135	227.343	211.574	323.618	171.324	142.252
	415.058	230.054	217.847	326.966	173.509	153.125
	902.801	587.152	589.227	748.301	498.397	489.099
	914.863	636.990	638.454	793.749	571.281	566.165
	1297.404	905.348	896.145	1144.466	802.836	801.952
	1411.179	952.155	972.300	1193.025	847.156	825.479
	1543.080	1065.365	1072.233	1378.895	972.410	950.893
	1766.843	1233.284	1277.700	1421.885	1042.853	1084.803
	1784.372	1246.257	1287.899	1473.743	1068.972	1100.454
	7864.661	7826.886	7847.631	7730.022	7747.265	7756.298
	7948.126	7926.369	7945.621	7833.453	7862.842	7871.222
	8150.293	8027.948	8059.192	7941.128	7908.270	7926.140
	8192.636	8131.564	8178.854	7990.573	8023.747	8067.599
	8255.602	8162.603	8212.629	8037.712	8041.310	8084.381
	8448.307	8268.844	8310.793	8282.155	8177.179	8199.736
	8456.183	8286.725	8324.144	8300.024	8200.042	8215.927
	…	…	…	…	…	…
3/2	24089.220	24878.442	25045.965	24025.489	24826.678	24924.821
	24089.769	24878.859	25050.191	24026.355	24829.323	24928.182
	24212.016	24938.925	25250.447	24098.607	24859.535	24953.101
	24216.611	24943.391	25276.157	24107.254	24867.875	24961.299
	24437.654	25142.728	25284.207	24319.195	25043.514	25138.292
	24457.534	25169.667	25307.902	24340.013	25061.376	25160.230
	24494.047	25172.825	25332.565	24379.566	25083.920	25173.699
	24523.677	25199.999	25360.688	24389.836	25099.942	25189.484
	24526.219	25220.129	25434.223	24402.741	25142.018	25248.532
	24540.219	25248.783	25442.568	24430.176	25177.239	25279.846
	24631.527	25326.897	25483.973	24515.567	25236.432	25334.083
	24673.884	25339.456	25496.477	24524.512	25269.491	25359.367
	24681.209	25378.389	25515.506	24553.713	25291.210	25385.392
	24721.840	25386.406	25549.553	24590.790	25326.020	25418.702
	24724.455	25408.220	25555.914	24604.023	25328.256	25421.254
	24750.429	25437.287	25564.352	24618.946	25338.147	25441.142
	24759.203	25443.368	25574.061	24637.113	25340.991	25444.941
	24773.311	25454.697	25600.410	24659.376	25375.470	25470.667
	…	…	…	…	…	…
1/2	36261.082	37383.117	37552.313	36120.008	37288.335	37437.764
	36263.201	37386.673	37556.093	36122.074	37291.596	37440.843
	36270.563	37394.315	37563.033	36125.220	37293.950	37443.636
	36273.153	37398.981	37567.972	36127.495	37297.703	37447.211
	36474.471	37557.726	37726.943	36322.087	37452.840	37604.295
	36485.801	37566.070	37734.584	36327.348	37454.787	37605.889
	36501.045	37571.716	37740.569	36343.198	37469.735	37620.570
	36504.919	37575.333	37744.192	36347.072	37473.026	37623.793
	36526.645	37615.652	37788.478	36377.230	37521.774	37678.168
	36577.834	37658.792	37832.372	36408.520	37547.097	37704.275
	36596.897	37690.621	37867.466	36454.019	37600.812	37763.060
	36614.602	37710.534	37889.328	36463.701	37605.239	37767.691
	36643.208	37744.925	37926.952	36504.200	37652.193	37818.028
	36677.199	37756.758	37935.608	36525.104	37661.801	37828.946

	36680.591	37766.668	37947.545	36533.498	37675.009	37842.896
	36713.633	37809.614	37984.848	36600.470	37728.882	37884.106
	36715.801	37811.426	37986.956	36601.855	37729.942	37885.495
	37784.444	38989.541	39160.535	37671.830	38905.462	39057.197
	37786.932	38995.480	39167.174	37677.426	38915.036	39068.446
	37861.250	39026.889	39196.933	37707.601	38922.724	39073.348
	37866.167	39036.762	39207.592	37716.807	38935.853	39088.201
	…	…	…	…	…	…

Table S3: Energies of the spin-orbit states (RASSI, cm^{-1}) obtained for compound 2

center Dy1			center Dy2 (closer to K)		
MB	VDZP	VTZP	MB	VDZP	VTZP
0.0	0.0	0.0	0.0	0.0	0.0
0.0	0.0	0.0	0.0	0.0	0.0
246.5	126.3	115.9	188.2	86.3	69.1
246.5	126.3	115.9	188.2	86.3	69.1
532.8	332.2	327.7	440.8	279.3	269.6
532.8	332.2	327.7	440.8	279.3	269.6
823.3	534.4	530.4	697.1	456.7	440.6
823.3	534.4	530.4	697.1	456.7	440.6
1084.2	721.6	720.3	932.7	626.2	614.0
1084.2	721.6	720.3	932.7	626.2	614.0
1278.1	856.9	851.3	1129.9	777.8	754.3
1278.1	856.9	851.3	1129.9	777.8	754.3
1478.2	987.6	1010.3	1254.0	855.6	853.6
1478.2	987.6	1010.3	1254.0	855.6	853.6
1720.0	1171.7	1213.7	1376.3	982.4	1021.0
1720.0	1171.7	1213.7	1376.3	982.4	1021.0
3542.3	3660.3	3638.0	3505.3	3641.7	3622.5
3542.3	3660.3	3638.0	3505.3	3641.7	3622.5
3816.8	3832.3	3806.3	3740.4	3784.9	3753.6
3816.8	3832.3	3806.3	3740.4	3784.9	3753.6
4087.3	4015.1	3986.2	3982.2	3949.9	3912.7
4087.3	4015.1	3986.2	3982.2	3949.9	3912.7
4296.8	4158.2	4125.8	4177.1	4080.6	4038.1
4296.8	4158.2	4125.8	4177.1	4080.6	4038.1
4448.0	4258.6	4228.4	4334.4	4187.2	4132.1
4448.0	4258.6	4228.4	4334.4	4187.2	4132.1
4657.2	4397.3	4389.7	4443.8	4267.8	4245.7
4657.2	4397.3	4389.7	4443.8	4267.8	4245.7
4969.3	4624.0	4634.3	4646.2	4445.6	4451.3
4969.3	4624.0	4634.3	4646.2	4445.6	4451.3
6084.7	6266.4	6233.4	6008.4	6224.0	6193.4
6084.7	6266.4	6233.4	6008.4	6224.0	6193.4
…	…	…	…	…	…

Table S4: Energies of the spin-free states (CASSCF, cm^{-1}) obtained for compound 3

Spin	center Er1			center Er2 (closer to K)		
	MB	VDZP	VTZP	MB	VDZP	VTZP
3/2	0.000	0.000	0.000	0.000	0.0	0.000
	3.111	4.869	3.639	6.519	7.7	4.239
	115.020	83.264	103.620	13.112	30.0	61.022
	115.532	90.264	109.525	19.018	39.3	67.175
	208.705	174.102	180.176	174.309	163.5	168.959
	225.265	178.531	185.630	196.966	170.1	177.354
	250.142	211.531	224.979	208.022	186.1	203.275
	331.753	235.651	243.624	283.585	210.3	215.495
	335.571	248.422	261.508	291.255	212.7	222.247
	615.665	470.409	487.767	542.054	434.5	453.861
	619.424	476.582	494.186	542.885	435.3	454.801
	741.919	547.178	563.698	618.887	477.9	493.705
	743.180	549.694	566.305	620.105	479.1	495.265
	17253.938	17958.383	18068.673	17239.930	17941.3	18061.140
	17255.091	17966.345	18074.171	17243.016	17952.1	18067.521
	17639.830	18186.170	18278.450	17642.016	18185.6	18270.283
	17728.391	18249.966	18338.134	17695.745	18233.6	18309.931

	17769.056	18277.684	18382.060	17755.403	18242.3	18345.953
	17796.354	18339.970	18449.867	17772.773	18337.9	18448.962
	18135.229	18530.618	18643.304	17999.665	18443.8	18554.713
	18245.623	18648.720	18764.638	18126.210	18579.4	18693.855
	27530.849	28380.737	28531.265	27474.428	28330.4	28474.845
	27553.281	28389.772	28542.028	27482.672	28341.5	28484.964
	27595.406	28402.192	28553.636	27503.386	28358.3	28510.501
	27788.547	28584.501	28740.514	27686.884	28515.0	28667.136
	27904.512	28701.883	28861.079	27809.020	28644.7	28802.327
	27957.205	28786.810	28949.534	27919.759	28760.5	28925.305
	28013.568	28806.147	28975.199	27924.083	28766.2	28931.057
	28040.107	28831.305	28994.709	27957.147	28789.1	28949.676
	28058.863	28854.509	29024.224	27965.354	28806.2	28975.011
	…	…	…	…	…	…
1/2	17292.960	17917.565	18047.449	17272.905	17900.137	18028.308
	17293.828	17918.150	18047.840	17273.474	17900.339	18028.644
	17359.103	17954.359	18079.612	17316.738	17923.701	18047.227
	17360.155	17955.560	18081.586	17317.281	17925.000	18049.450
	17436.118	17999.013	18122.387	17389.535	17968.823	18091.160
	17444.193	18004.287	18128.581	17390.593	17970.177	18092.616
	17486.853	18027.891	18148.572	17437.897	17996.689	18114.068
	17512.791	18047.999	18171.694	17442.450	18005.342	18128.631
	17521.760	18053.603	18176.005	17461.098	18015.889	18136.485
	17590.885	18099.097	18222.979	17497.246	18043.753	18168.278
	17591.059	18099.310	18223.231	17497.878	18043.895	18168.440
	22684.858	23581.286	23735.519	22690.617	23581.266	23737.894
	22686.390	23583.902	23738.691	22690.776	23583.034	23741.053
	22984.278	23758.116	23905.564	22934.928	23724.486	23868.703
	22999.233	23774.992	23921.506	22952.129	23742.978	23888.024
	23207.804	23917.549	24057.737	23151.279	23882.481	24029.039
	23249.583	23942.855	24094.834	23188.001	23900.294	24034.078
	23320.529	23988.260	24132.885	23267.761	23951.322	24084.377
	23531.999	24145.694	24307.567	23374.910	24055.665	24217.778
	23533.754	24148.034	24310.608	23379.610	24056.240	24220.339
	25264.357	26378.113	26519.679	25424.708	26462.900	26593.309
	25265.522	26378.425	26519.895	25431.372	26463.937	26593.646
	25560.670	26619.260	26782.028	25551.428	26617.055	26803.143
	25586.694	26635.600	26793.250	25603.612	26649.577	26830.279
	25721.001	26722.130	26911.222	25663.649	26676.372	26865.063
	25850.257	26798.002	26984.473	25795.742	26753.362	26937.043
	25884.486	26841.710	27016.340	25857.828	26828.239	27004.424
	26146.747	27001.672	27177.492	26088.933	26968.171	27138.390
	26166.878	27030.175	27207.926	26101.906	26987.275	27167.555
	26519.826	27257.286	27429.725	26419.012	27193.587	27358.130
	…	…	…	…	…	…

Table S5: Energies of the spin-orbit states (RASSI, cm^{-1}) obtained for compound 3

center Er1			center Er2 (closer to K)		
MB	VDZP	VTZP	MB	VDZP	VTZP
0.0	0.0	0.0	0.0	0.0	0.0
0.0	0.0	0.0	0.0	0.0	0.0
130.9	99.6	121.5	12.4	37.7	73.9
130.9	99.6	121.5	12.4	37.7	73.9
173.1	142.2	148.3	138.1	130.3	138.4
173.1	142.2	148.3	138.1	130.3	138.4
237.5	198.8	211.0	177.7	168.5	188.0
237.5	198.8	211.0	177.7	168.5	188.0
318.5	247.3	261.5	259.6	214.4	231.1
318.5	247.3	261.5	259.6	214.4	231.1
440.8	323.5	334.7	366.3	276.1	285.1
440.8	323.5	334.7	366.3	276.1	285.1
662.1	508.0	525.2	563.6	454.9	475.7
662.1	508.0	525.2	563.6	454.9	475.7
751.2	557.7	573.0	618.8	483.1	500.5
751.2	557.7	573.0	618.8	483.1	500.5
6342.5	6640.4	6594.9	6328.7	6634.0	6590.4
6342.5	6640.4	6594.9	6328.7	6634.0	6590.4
6445.3	6731.1	6699.2	6354.0	6678.6	6664.0
6445.3	6731.1	6699.2	6354.0	6678.6	6664.0

6488.9	6757.1	6722.5	6435.1	6732.1	6698.1
6488.9	6757.1	6722.5	6435.1	6732.1	6698.1
6573.1	6823.6	6789.9	6508.5	6787.8	6759.7
6573.1	6823.6	6789.9	6508.5	6787.8	6759.7
6691.0	6901.7	6866.9	6617.3	6856.6	6823.3
6691.0	6901.7	6866.9	6617.3	6856.6	6823.3
6868.4	7041.3	7010.3	6775.7	6989.0	6962.0
6868.4	7041.3	7010.3	6775.7	6989.0	6962.0
6973.3	7100.4	7066.3	6852.2	7028.9	6996.5
6973.3	7100.4	7066.3	6852.2	7028.9	6996.5
10259.3	10710.8	10665.3	10227.0	10694.2	10652.4
10259.3	10710.8	10665.3	10227.0	10694.2	10652.4
…	…	…	…	…	…

Table S6: Energies of the spin-free states (CASSCF, cm^{-1}) obtained for compound 4

Spin	center Er1 (closer to K)		center Er2	
	MB	VTZP	MB	VTZP
3/2	0.000	0.000	0.000	0.000
	4.289	3.971	3.278	4.644
	11.047	55.444	78.646	76.283
	15.708	60.947	79.520	81.932
	164.763	164.963	191.388	181.490
	185.434	173.560	218.862	187.682
	209.498	196.178	231.046	208.715
	282.097	213.211	322.865	241.982
	290.020	220.784	326.843	248.471
	541.452	449.134	612.684	482.300
	543.130	452.390	613.781	484.145
	632.411	499.354	775.525	581.542
	633.834	501.969	775.934	582.357
	17223.296	18045.865	17191.770	18026.327
	17226.649	18052.120	17193.894	18032.905
	17638.290	18274.982	17630.826	18288.316
	17694.880	18324.399	17719.116	18376.710
	17754.726	18345.399	17768.964	18378.526
	17778.286	18438.286	17835.010	18443.366
	18012.271	18558.975	18149.643	18640.460
	18129.892	18697.099	18245.684	18766.845
	27486.646	28484.081	27542.082	28529.310
	27495.699	28493.087	27555.129	28551.803
	27513.128	28520.379	27593.624	28557.443
	27703.935	28679.509	27810.496	28754.126
	27818.730	28809.408	27929.105	28879.216
	27906.946	28918.089	27945.863	28943.805
	27914.418	28918.681	27978.126	28950.970
	27952.200	28944.944	28019.763	28978.911
	27962.976	28966.223	28059.603	29019.666
	…	…	…	…
1/2	17265.233	18023.205	17273.547	18035.533
	17265.754	18023.429	17274.152	18035.933
	17315.335	18045.826	17353.320	18076.984
	17316.181	18048.458	17353.590	18078.798
	17388.200	18089.072	17437.498	18123.360
	17391.558	18092.943	17439.879	18126.899
	17438.405	18116.237	17495.183	18153.414
	17446.860	18128.474	17505.539	18167.374
	17464.103	18136.694	17519.597	18173.113
	17495.280	18168.386	17575.671	18215.887
	17496.265	18168.449	17575.958	18216.071
	22676.350	23726.109	22646.659	23709.474
	22677.330	23728.745	22647.261	23711.162
	22938.901	23871.372	22989.981	23910.107
	22955.784	23891.298	23007.480	23929.275
	23154.372	24030.040	23239.419	24084.649
	23202.963	24046.468	23259.128	24096.172
	23281.885	24094.948	23350.788	24152.340

	23375.581	24220.846	23506.782	24296.385
	23383.195	24222.312	23511.404	24297.051
	25416.021	26587.281	25288.450	26533.507
	25424.433	26587.837	25291.270	26534.043
	25522.609	26784.141	25511.306	26762.373
	25567.855	26812.561	25572.436	26793.569
	25659.612	26847.132	25609.953	26832.520
	25784.139	26920.471	25775.198	26921.135
	25845.175	26995.745	25829.973	26989.127
	26081.222	27133.520	26108.544	27156.314
	26092.526	27159.819	26123.561	27179.356
	26420.169	27363.981	26509.243	27428.644
	…	…	…	…

Table S7: Energies of the spin-orbit states (RASSI, cm^{-1}) obtained for compound 4:

center Er1 (closer to K)		center Er2	
MB	VTZP	MB	VTZP
0.0	0.0	0.0	0.0
0.0	0.0	0.0	0.0
7.1	67.8	87.6	88.1
7.1	67.8	87.6	88.1
130.2	136.3	162.1	149.1
130.2	136.3	162.1	149.1
174.7	181.9	216.6	199.0
174.7	181.9	216.6	199.0
257.7	228.3	304.1	248.5
257.7	228.3	304.1	248.5
367.2	288.9	433.7	331.6
367.2	288.9	433.7	331.6
567.1	476.4	663.9	522.9
567.1	476.4	663.9	522.9
633.0	507.5	783.6	589.3
633.0	507.5	783.6	589.3
6325.9	6589.6	6340.8	6594.8
6325.9	6589.6	6340.8	6594.8
6348.3	6655.9	6410.0	6673.7
6348.3	6655.9	6410.0	6673.7
6431.5	6696.1	6470.3	6712.3
6431.5	6696.1	6470.3	6712.3
6506.1	6756.7	6555.8	6778.5
6506.1	6756.7	6555.8	6778.5
6617.3	6825.2	6681.6	6862.0
6617.3	6825.2	6681.6	6862.0
6778.6	6962.6	6868.7	7007.7
6778.6	6962.6	6868.7	7007.7
6865.7	7004.8	7001.1	7081.9
6865.7	7004.8	7001.1	7081.9
10223.5	10651.2	10253.2	10663.6
10223.5	10651.2	10253.2	10663.6
…	…	…	…

All states correspond to the 4f-4f transitions, i.e. are of ligand field type. No charge transfer states were considered. For the modeling of the exchange interaction between Ln sites the VTZP results were employed.

Analysis of the multiplet-specific crystal-field for Dy sites in 1 and 2

Recently, the extraction of the parameters of the multiplet-specific crystal-field for lanthanides methodology has been implemented in the SINGLE_ANISO program in MOLCAS. The results presented below use the ab initio CASSCF/RASSI wave function and energies to compute the parameters of the crystal-field splitting of the ground *J* multiplet.
The Crystal-Field Hamiltonian:

$$H_{CF} = \sum_{k,q} B_k^q O_k^q$$

where:

O_k^q -- Extended Stevens Operators (ESO) as defined in:

1. Rudowicz, C.; J. Phys. C: Solid State Phys.,18 (1985) 1415-1430.
2. Implemented in the "EasySpin" function in MATLAB, www.easyspin.org.

k - the rank of the ITO, = 2, 4, 6.

q - the component (projection) of the ITO, = $-k$, $-k+1$, ... 0, 1, ... k;

Quantization axis was chosen the main magnetic axis of the ground Kramers doublet.

Table S8. Parameters of the *ab initio* crystal field for the individual Ln sites in compounds **2-4**. (TZP basis)

Rank	Proj.	Compound 2		Compound 3		Compound 4	
k	q	Dy1	Dy2	Er1	Er2	Er1	Er2
2	-2	-0.535140E-01	-0.140417E+00	-0.546539E+00	0.194653E-01	-0.189670E+00	-0.202116E-03
	-1	0.201439E+01	-0.837609E+00	-0.371786E+00	0.126786E+00	0.401697E+00	-0.248221E+00
	0	-0.551468E+01	-0.471389E+01	-0.244231E+01	-0.204053E+01	-0.205323E+01	-0.236264E+01
	1	0.231254E+00	-0.468048E+00	-0.269014E+00	-0.417252E-01	0.228275E-01	-0.964296E-01
	2	0.649035E+01	0.518941E+01	0.231406E+01	0.218899E+01	0.244990E+01	0.311724E+01
4	-4	-0.467820E-02	-0.841583E-02	0.126499E-01	0.268865E-02	0.860376E-02	-0.257562E-02
	-3	-0.709719E-01	0.278916E-01	-0.580828E-02	0.190522E-02	-0.101570E-02	0.307589E-02
	-2	-0.117192E-02	-0.427150E-02	0.288370E-02	0.513570E-03	0.160784E-02	-0.615698E-03
	-1	-0.287663E-01	0.141000E-01	0.269479E-02	-0.596242E-03	-0.167835E-02	0.944818E-03
	0	0.292096E-02	0.518216E-02	0.269434E-03	-0.144163E-03	-0.940719E-04	0.911350E-04
	1	0.407627E-02	0.109271E-02	0.355323E-03	-0.126146E-03	0.122872E-02	0.430922E-03
	2	0.423533E-01	0.483016E-01	-0.192298E-01	-0.178598E-01	-0.178150E-01	-0.201671E-01
	3	0.757695E-02	0.744148E-02	0.101029E-01	-0.784807E-03	-0.240173E-02	0.157817E-01
	4	0.302131E-01	0.338922E-01	-0.415297E-01	-0.471105E-01	-0.442765E-01	-0.407549E-01
6	-6	0.262912E-05	-0.173971E-07	0.811522E-04	0.136673E-04	0.386364E-04	-0.397995E-05
	-5	-0.352270E-03	0.114006E-03	-0.124379E-03	0.171786E-05	-0.160369E-03	0.983794E-04
	-4	-0.252467E-05	-0.158880E-04	-0.842563E-04	-0.156848E-04	-0.350193E-04	0.157681E-04
	-3	0.598729E-04	-0.857549E-05	0.971150E-04	-0.426555E-04	-0.484972E-04	-0.510964E-05
	-2	0.299320E-05	0.767148E-05	0.151308E-04	0.248996E-05	-0.132274E-05	0.469280E-05
	-1	0.662807E-04	-0.420457E-04	-0.158505E-04	0.191147E-04	0.384596E-05	0.967831E-05
	0	-0.178829E-05	-0.595496E-05	-0.207138E-04	-0.197021E-04	-0.194103E-04	-0.210516E-04
	1	-0.688711E-04	0.240167E-04	0.292318E-04	0.166737E-04	-0.304174E-04	0.371577E-06
	2	-0.115581E-03	-0.104506E-03	0.518185E-04	0.982957E-05	0.196359E-04	0.665648E-04
	3	-0.700622E-05	-0.463034E-04	-0.750018E-04	-0.797069E-04	0.456875E-04	-0.163746E-03
	4	0.120817E-03	0.114721E-03	0.187624E-03	0.188058E-03	0.170024E-03	0.162188E-03
	5	0.672395E-04	-0.540420E-04	0.122807E-03	-0.122831E-03	0.106352E-03	0.310352E-03
	6	0.135509E-04	0.856097E-06	-0.556321E-04	-0.985607E-04	-0.124296E-03	-0.171449E-03

References

1. Yasuda, H.; Ohnuma, Y.; Yamauchi, M.; Tani, H.; Nakamura, A., *Bulletin of the Chemical Society of Japan* **1979,** *52* (7), 2036-2045.
2. Bradley, D. C.; Ghotra, J. S.; Hart, F. A., *Journal of the Chemical Society-Dalton Transactions* **1973,** (10), 1021-1027; Schuetz, S. A.; Day, V. W.; Sommer, R. D.; Rheingold, A. L.; Belot, J. A., *Inorganic Chemistry* **2001,** *40* (20), 5292-+.
3. v.2012, A. S. S.; Bruker AXS: Madison, W., 2005.
4. Blessing, R. H., *Acta Crystallographica Section A* **1995,** *51*, 33-38.
5. Sheldrick, G. M., *Acta Crystallographica Section A* **2008,** *64*, 112-122.
